# Four New Furofuran Lignans from *Phryma leptostachya* Inhibit the Accumulation of Molting Hormones in Armyworm

**DOI:** 10.3390/ijms25137081

**Published:** 2024-06-27

**Authors:** Jiaming Zhang, Qi Cong, Yuyao Sun, Juan Hua, Shihong Luo

**Affiliations:** Engineering Research Center of Protection and Utilization of Plant Resources, College of Bioscience and Biotechnology, Shenyang Agricultural University, Shenyang 110866, China

**Keywords:** lignans, *Phryma leptostachya*, *Mythimna separata*, antifeedant, molting hormones

## Abstract

Furofuran lignans have been identified as the main substances responsible for the biological activities of the plant genus *Phryma*. Here, four new phrymarolin-type leptolignans A–D (**7**–**10**) and eight previously known lignans were isolated from *P. leptostachya*. Of these, nine exhibited significant antifeedant activity against armyworm (*Mythimna separata*) through a dual-choice bioassay, with the EC_50_ values ranging from 0.58 to 10.08 μg/cm^2^. In particular, the newly identified lignan leptolignan A (**7**) showed strong antifeedant activity, with an EC_50_ value of 0.58 ± 0.34 μg/cm^2^. Further investigation found that leptolignan A can inhibit the growth and nutritional indicators in the armyworm *M. separata*. The concentrations of two molting hormones, 20-hydroxyecdysone and ecdysone, were also found to decrease significantly following the treatment of the armyworms with the lignan, implying that the target of the *P. leptostachya* lignan may be involved in 20-hydroxyecdysone and ecdysone synthesis. These results enrich our knowledge of *P. leptostachya* metabolite structural diversity, and provide a theoretical basis for the control of armyworm using lignans.

## 1. Introduction

*Mythimna separata* (armyworm) is a notorious phytophagous insect in the Lepidoptera family, and mainly targets maize, wheat, and rice [1,2]. This highly destructive pest can migrate seasonally over long distances and is widely distributed throughout Asia and Australia [3]. It has been reported that almost 1700 million hectares of farmland can be threatened by *M. separata* every year [4]. The pyrethroid pesticide lambda-cyhalothrin is typically used to control armyworms, but places great stress on the environment [5]. Recent research has suggested a series of essential oils which have anti-armyworm activity and which will soon be used in the field [6]. In addition, genes which interfere with the night migration of *M. separata* have been used as effective molecular targets for integrated armyworm management [2,7]. However, although various control strategies have been proposed, *M. separata* remain difficult to control effectively due to their long-distance migration and high reproductive capacity. There is therefore still a long way to go in the prevention and control of this harmful agricultural pest.

Plants are immobile and usually respond to external stresses through trade-offs between primary or specialized metabolites involved in growth and defense [8]. Discovering new natural products with biological activities has always been an intriguing research topic, and plant specialized defense metabolites are a rich source of potential natural pesticides [9,10]. *Phryma leptostachya* is a perennial herb, and is used in Chinese Traditional Medicine as a remedy for rheumatism [11]. Under the food security strategy, many plants with biological activity have been investigated as potential natural insecticides in an attempt to reduce the environmental damage and threat to human health posed by conventional pesticides. Because of its role in TCM, *P. leptostachya* was investigated and has since been developed into an insecticide [12]. Previous phytochemical studies have revealed that the main active substances in this species are furofuran lignans, which have been found to have insecticidal activities against *Musca domestica*, *Aedes albopictus*, and *A. aegypti* [13,14]. However, the synthesis and accumulation of these active plant specialized metabolites are known to be greatly affected by factors related to the external environment, including temperature and altitude [15,16]. The *P. leptostachya* individuals used in most current studies are grown in Shanxi Province, China, which has a temperate continental climate [11,12,13]. To explore the active substances in plants grown under different conditions, we therefore systematically isolated and identified the metabolites in whole *P. leptostachya* plants grown in Shenyang City, which has a temperate monsoon climate. We used chromatographic isolation and spectroscopic data analysis to isolate and identify twelve lignans from *P. leptostachya.* Two skeleton types (leptostachyol acetate-type and phrymarolin-type) were identified in this study. The compounds were assessed for antifeedant activity against *M. separata* and compound **1** was used to explore the mechanism of action of the biological activity of these lignans against armyworm.

## 2. Results and Discussion

### 2.1. Elucidation of the Structures of the Isolated Compounds

**Figure 1:**
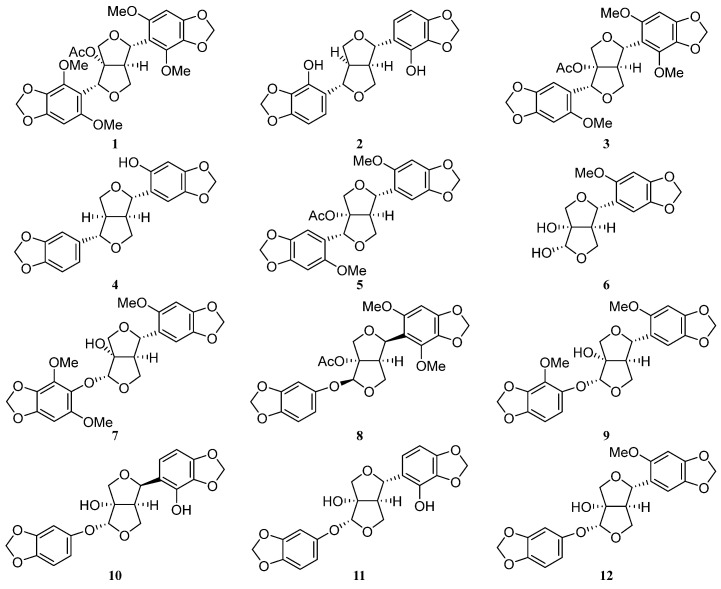
Chemical structures of compounds **1**–**12** isolated from whole *P. leptostachya* plants.

The main active metabolites from sun-dried whole *P. leptostachya* plants grown in Shenyang City were traced and isolated. Twelve target compounds (including four new lignans and eight previously known lignans) were successfully isolated and identified (Figure 1). After comparing their physical and spectral data with published values, the known compounds were identified as leptostachyol acetate Ⅰ (**1**) [17], sesamin-2,2′-diol (**2**) [18], 8′-acetoxy-2,2′,6-trimethoxy-3,4,4′,5′-methylenedioxyphenyl-7,7′-dioxabicyclo[3.3.0] octan (**3**) [17], sesaminol (**4**) [19], (+)-phrymarin Ⅰ (**5**) [20], (1S,2S,5R,6S)-1,2-dihydroxy-6-(2-methoxy-4,5-methylenedioxy phenyl)-3, 7-dioxabicyclo[3.3.0]octane (**6**) [21], phrymarolin III (**11**) [22], and 1-hydroxy-6*β*-(2′-methoxy-4′,5′-methylenedioxyphenyl)-2*β*-(3″,4″-methylenedioxyphenyl)oxy-3,7-dioxabicyclo-[3.3.0]octane (**12**) [23].

**Figure 2:**
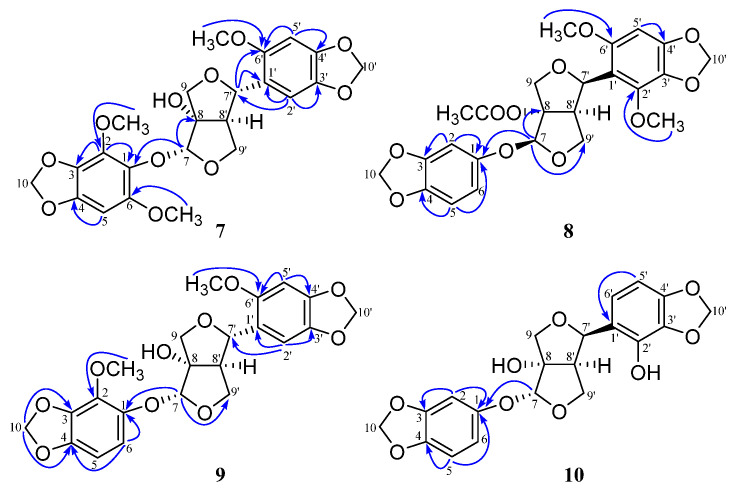
Significant HMBC correlations (H/C) of compounds **7**–**10**.

Compound **7** was obtained as a white solid. Its molecular formula was determined to be C_23_H_24_O_11_ from the HR-ESI-MS (*m*/*z* 499.1208 [M + Na]^+^, calculated for C_23_H_24_NaO_11_, 499.1211) and ^13^C NMR data. The ^1^H NMR spectrum of **7** (Table 1) exhibited the characteristic resonances of oxygenated methines and methylenes at *δ*_H_ 5.11 (1H, s, H-7), *δ*_H_ 4.69 (1H, d, *J* = 6.1 Hz, H-7’), 4.44 (1H, dd, *J* = 7.6, 8.8 Hz, H-9’a), 4.07 (1H, d, *J* = 11.5 Hz, H-9a), 3.81 (1H, d, *J* = 2.4 Hz, H-9’b), and 3.48 (1H, d, *J* = 11.5 Hz, H-9b). The ^1^H NMR spectrum also showed two dioxymethylene signals at *δ*_H_ 5.81 and 5.79 (each 2H, m), and three methoxy group signals at *δ*_H_ 3.82, 3.68, and 3.65 (each 3H, s). The ^13^C NMR and DEPT spectral data (Table 2) exhibited 23 carbon resonances, including three methoxy groups, four methylenes, six methines, and ten quaternary carbons, including one oxygenated carbon (*δ*_C_ 93.4), and nine olefinic carbons (*δ*_C_ 152.1, 149.0, 148.0, 145.5, 142.0, 139.2, 132.4, 132.2, and 124.0). These data were found to be similar to a previously reported furofuran lignin, compound **12**, containing a *cis*-3,7-dioxabicyclo[3.3.0]octane skeleton [20]. The main difference between them was that **7** had two more methoxy signals at *δ*_C_ 60.4 and 56.7. In the HMBC spectrum of **7**, the simultaneous ^1^H-^13^C correlations from *δ*_H_ 3.82 to *δ*_C_ 139.2, and *δ*_H_ 3.65 to *δ*_C_ 149.0, indicated that the methoxy groups were assignable to C-2 and C-6, respectively (Figure 2). The ROESY spectrum of **7** (Figure S6) indicated that the relative configurations of the stereogenic centers in **7** were the same as those in **12**. Therefore, compound **7** was identified as leptolignan A.

Compound **8** was obtained as a white solid. Its molecular formula was determined to be C_24_H_24_O_11_ from the HR-ESI-MS (*m*/*z* 511.1220 [M + Na]^+^, calculated for C_24_H_24_NaO_11_, 511.1211) and ^13^C NMR data. The ^1^H NMR data of compound **8** displayed signals similar to those of phrymarolin I [12]. The ^13^C NMR data (Table 2) displayed 24 carbon signals with three methyls (two signals for methoxyl groups at *δ*_C_ 57.2 and 60.4, and one signal for an acetyl group at *δ*_C_ 21.1), four methylenes, seven methines (four olefinic carbon signals at *δ*_C_ 110.9, 108.6, 104.3, and 101.5), and ten quaternary carbons, including an ester carbonyl carbon signal at *δ*_C_ 171.3. The main difference between these two lignans was the position of the methoxy group. The HMBC spectrum showed the connectivity of H-7′ (*δ*_H_ 4.96) to C-8′, C-9′, C-1′, and *δ*_C_ 144.0, and a methoxyl signal at *δ*_H_ 3.96 to *δ*_C_ 144.0. These data indicated that the methoxyl signal at *δ*_H_ 3.96 in **8** was linked to C-2′ (Figure 2). In the ROESY experiment, H-9b correlated with H-7, H-7′, and H-8′, which confirmed that H-7, H-7′, and H-8′ were in an *α* configuration. The other relative configurations of the stereogenic centers in **8** were the same as those in phrymarolin I, and thus, compound **8** was identified as leptolignan B.

Compound **9** was obtained as a white solid. Its molecular formula was determined to be C_22_H_22_O_10_ from the HR-ESI-MS (*m*/*z* 469.1113 [M + Na]^+^, calculated for C_22_H_22_NaO_10_, 469.1105) and ^13^C NMR data. The ^1^H NMR and ^13^C NMR data showed similar signals to those of compound **8** (Table 1 and Table 2), but no methyl signal at *δ*_H_ 2.16 (3H, s) and no carbonyl carbon signal at *δ*_C_ 169.8 (COCH_3_). These 1D NMR spectra of **9** indicated that the oxygenated quaternary carbon signal at C-8 was a hydroxyl substitution. The HMBC spectrum indicated that the methoxy proton at *δ*_H_ 3.83 correlated to *δ*_C_ 152.1, and *δ*_H_ 3.96 to *δ*_C_ 137.2. The methine proton resonances at *δ*_H_ 4.86 (1H, d, *J* = 6.1 Hz, H-7′) also showed correlations with C-1′, C-2′, C-8′, C-9′, and the oxygenated olefinic quaternary carbon signal at *δ*_C_ 152.1. The olefinic proton signal at *δ*_H_ 6.68 (1H, d, *J* = 8.4 Hz, H-6) correlated to C-1, C-4, C-3, and *δ*_C_ 137.2, and these data therefore indicated that two methoxyl groups (*δ*_H_ 3.83 and 3.96) were linked to C-6′ and C-2, respectively (Figure 2). The observation of the ROESY correlations of H-7 with H-9b and H-7′, and H-8′ with H-9a, indicated that H-7, H-9b, and H-7′ were in the same orientation (*β* configuration), and that H-8′ and H-9a were in an *α* configuration. Therefore, compound **9** was identified as leptolignan C.

Compound **10** was obtained as a white solid. Its molecular formula was determined to be C_20_H_18_O_9_ from the HR-ESI-MS (*m*/*z* 425.0837 [M + Na]^+^, calculated for C_20_H_18_NaO_9_, 425.0843) and ^13^C NMR data. The ^1^H NMR and ^13^C NMR data showed similar signals to those of the known compound **11** (Table 1 and Table 2) [22]. Analysis of the above three compounds showed that the relative configuration of C-7′ significantly affected the methine signal at C-7′ in the ^13^C NMR data. The signal at C-7′ (*δ*_C_ 80.5) in **10** was similar to that (*δ*_C_ 81.1) in **8**, and lower than those (*δ*_C_ 85.0) in **7** and **9**, indicating that the relative configuration of C-7′ in **10** and **8** may be the same. Furthermore, the correlation of H-7′ and H-8′ in the ROESY spectrum confirmed that H-7′ and H-8′ in **10** were in an *α* configuration. The other relative configurations of the stereogenic centers in **10** were same as those in **11**. Therefore, compound **10** was identified as leptolignan D.

### 2.2. Antifeedant Activity

**Figure 3:**
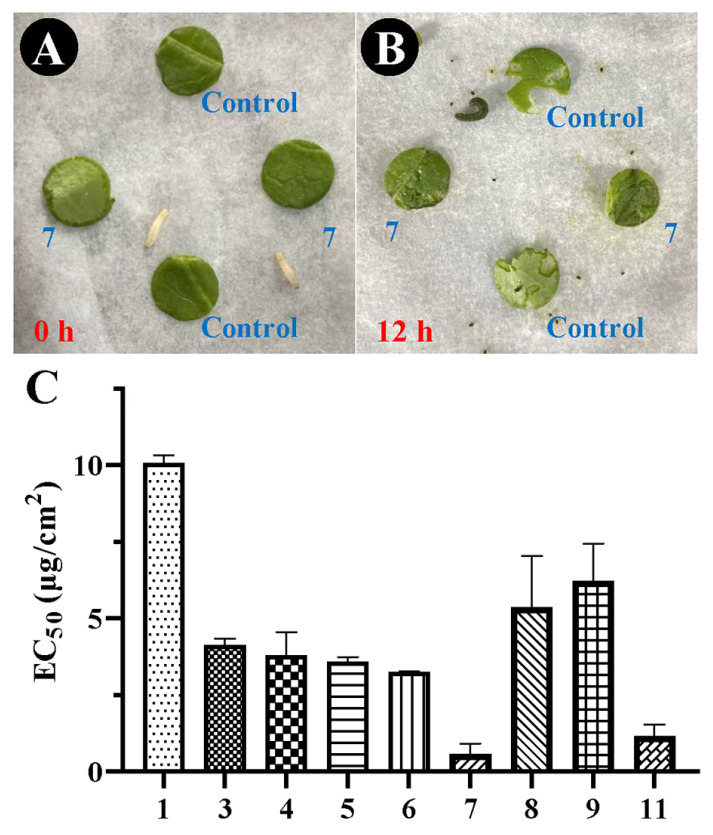
The antifeedant activity of the compounds isolated from whole *P. leptostachya* plants against *M. separata* larvae. Antifeedant assay of compound **7** following 0 (**A**) and 12 (**B**) h treatment. The EC_50_ values of compounds **1**, **3**–**9**, and **11** (**C**). Control, leaf disc treated with 10 μL acetone; **7**, leaf disc treated with 10 μL compound **7** (dissolved in acetone).

The antifeedant activity of the isolated compounds **1**, **3**–**9**, and **11** against second-instar *M. separata* larvae was investigated. All tested lignans exhibited moderate antifeedant activity, with EC_50_ values ranging from 0.58 to 10.08 μg/cm^2^ (Figure 3). Of the tested compounds, the phrymarolin-type lignan leptolignan A (**7**) showed the strongest antifeedant activity, with an EC_50_ value of 0.58 ± 0.34 μg/cm^2^, followed by the phrymarolin-type lignan **11**, with an EC_50_ value of 1.17 ± 0.37 μg/cm^2^. Their antifeedant activity levels were both significantly stronger than that of commercial neem oil, which has an EC_50_ value of 1.68 ± 0.11 μg/cm^2^, while compound **1** showed less potent antifeedant activity than leptolignan A, with an EC_50_ value of 10.08 ± 0.25 μg/cm^2^.

### 2.3. Growth Inhibition Activity

**Figure 4:**
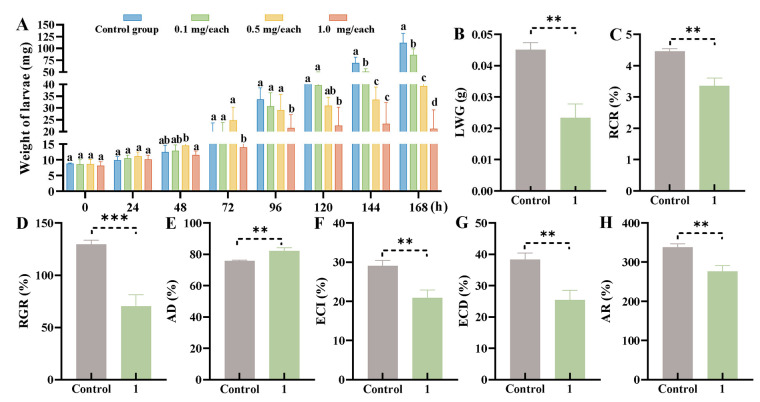
Growth-inhibiting activity of compound **1** against *M. separata* larvae. (**A**) The weight of each larva fed with forage containing different concentrations of compound **1**. (**B**–**H**) The nutritional indexes of *M. separata* larvae following 72 h treatment with compound **1**. RCR, relative consumption rate; RGR, relative growth rate; LWG, larval weight gain; AD, approximate digestibility; AR, assimilation rate; ECI, efficiency of conversion of ingested food to body substance; and ECD, efficiency of conversion of digested food into growth. Data are expressed as mean ± standard deviation. Independent-samples *t* tests were used to analyze differences between two groups, ** (*p* < 0.01), *** (*p* < 0.001), and one-way ANOVA with Tukey’s tests were used to analyze differences among four groups. Data labeled with the same letter show no significant differences (*p* > 0.05).

The growth inhibition activity of compound **1** against *M. separata* was used to further investigate the mechanism underlying the anti-insect activity of lignans. Forage was prepared containing compound **1** dissolved in acetone to reach final concentrations of 1, 0.5, 0.1, or 0 mg/each, and each *M. separata* larva (second instar) was allowed to feed for 7 days. Following 72 h treatment with compound **1** at 1 mg/each, the larval weights were found to be significantly lower compared with those of the control group (*p* < 0.001) (Figure 4A). Following 144 h treatment, all concentrations of compound **1** exhibited conspicuous growth inhibition effects on *M. separata* larvae, producing weight inhibition ratios of 61.64 ± 16.11%, 50.12 ± 11.86%, and 24.92 ± 14.26% at concentrations of 1, 0.5, and 0.1 mg/each, respectively. Compound **1** at 1 mg/each reached its highest inhibitory activity at 168 h, with an inhibition ratio of 80.32 ± 5.35%. These results suggested that compound **1** can significantly inhibit the growth of *M. separata*.

Calculation of the nutritional indexes of larvae feeding for 72 h on forage treated with different concentrations of compound **1** showed that the larval weight gain (LWG), relative consumption rate (RCR), relative growth rate (RGR), efficiency of conversion of ingested food to body substance rate (ECI), efficiency of conversion of digested food into growth rate (ECD), and assimilation rate (AR) were 0.023 ± 0.004, 3.36 ± 0.25%, 70.35 ± 10.97%, 20.86 ± 2.06%, 25.42 ± 3.12%, and 276.04 ± 15.18%, respectively (Figure 4B–D, and 4F–H). All indexes were significantly reduced compared with those of the control group (*p* < 0.005). However, the approximate digestibility increased markedly to 7.79 ± 2.56% (*p* < 0.005, Figure 4E). These results suggested that compound **1** was able to interfere with the nutritional indexes in *M. separata* and therefore inhibit its growth.

The reactions of insects to plant metabolites is very important for the management of pests [23]. Toxic compounds like 7-dehydroabietanone can significantly change the physiological and biochemical indicators of insects, and can even activate genes related to detoxification [24]. The change in weight and nutritional indexes in *M. separata* following the consumption of compound **1** suggest changes in a series of related enzymes in *M. separata*, and investigating these related target enzymes may be helpful to promote the use of lignans in the integrated control of armyworm.

### 2.4. Detection of Hormones in M. separata Larvae

**Figure 5:**
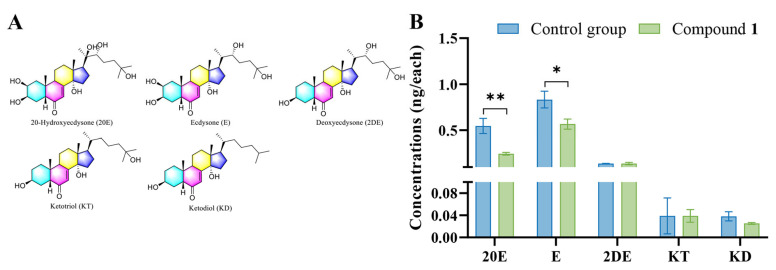
Quantitative analysis of the ecdysteroids produced in *M. separata* larvae following treatment with compound **1**. (**A**) Chemical structures of *M. separata* ecdysteroids. (**B**) The concentrations of the ecdysteroids in *M. separata* larvae following treatment with compound **1**. Data are expressed as mean ± standard deviation, and independent-samples *t* tests were used to analyze statistical differences, * (*p* < 0.05); **(*p* < 0.01).

Molting hormones are critical for insect growth. Five molting hormones in *M. separata* larvae were detected through UPLC-MS/MS following 72 h treatment with compound **1** at a concentration of 1 mg/g. In the treatment group, the concentrations of two molting hormones, 20-hydroxyecdysone and ecdysone, decreased absolutely, with values of 0.24 ± 0.014 and 0.56 ± 0.055 ng/each, respectively (Figure 5). However, there were no obvious changes in the concentrations of three precursor enzymes between the treatment and control groups. The variation in levels of these two molting hormones between the treatment and control groups, therefore, suggested that the biosynthesis pathway of ecdysone and 20-hydroxyecdysone in *M. separata* larvae may be influenced by compound **1**. Ecdysone and 20-hydroxyecdysone are important endogenous hormones that regulate growth, molting, and metamorphosis in insects [25], and 20-hydroxyecdysone has also been shown to control the synthesis of glycogen in silkworm, affecting its nutritional status [26]. In this study, the reduction of 20-hydroxyecdysone in armyworms was accompanied by a decrease in six nutritional indexes. These results suggested that lignans may act against armyworms through affecting the normal synthesis of these molting hormones. Future studies investigating the mechanism by which plant lignans act against *M. separata* should therefore focus on the process of ecdysone and 20-hydroxyecdysone synthesis.

## 3. Materials and Methods

### 3.1. General Experimental Procedures

Column chromatography was performed on a 200-mesh silica gel, Sephadex LH-20, or MCI gel CHP-20P (75–150 μm) with different solvent systems, and the collected fractions were spotted on glass precoated TLC silica gel GF_254_ plates. After treatment with ultraviolet light (254 nm) and spraying with 15% H_2_SO_4_ developing solution, TLC spots then appeared. ^1^H and ^13^C nuclear magnetic resonance (NMR) spectra were recorded at 600 and 150 MHz (Bruker AVANCE 600 MHz) (Bruker, Karlsruhe, Germany) in acetone-*d*_6_ with TMS as the internal standard. The mass spectrometry (MS) data were measured using Agilent technology with a version of Q-Tof B.05.01 (B5125.2). UV Spectra: SHIMADZU UV-2401PC, MeOH; λ_max_ (log *ε*) in nm. IR Spectra: Bruker Vertex 70, KBr tablet.

### 3.2. Plant Material

Whole plants of *P. leptostachya* were collected in June 2021 from the Oak Germplasm Repository of Shenyang Agricultural University (E: 123°33′, N: 41°49′), Liaoning Province, China, after morphological identification by Professor Bo Qu. A voucher specimen of *P. leptostachya* was deposited at the Research Center of Protection and Utilization of Plant Resources, Shenyang Agricultural University, under the voucher number of SYNUB015936–SYNUB015939.

### 3.3. Extraction and Isolation

After air-drying, whole plants of *P. leptostachya* (6.0 kg) were crushed and then soaked in methanol for 24 h. A rotary evaporator was used to dry the methanol extracts and the samples were then subjected to ethyl acetate to obtain the organic phase (50 g). The organic phase was subsequently mixed with 200-mesh silica gel, and then eluted on a silica gel column with a chloroform–acetone (9:0–0:9) solvent system to give 5 fractions (fractions 1–5). Fraction 2 was subjected to MCI gel column chromatography with methanol–water (80–100%) to afford 3 subfractions (fractions 2a–2c). Subfraction 2b (methanol/water, 90%) was purified on a Sephadex LH-20 (acetone as eluent) column and semi-preparative HPLC (C_18_ column; detected at 230 nm) and eluted with methanol–water (80–100%) to yield compounds **2** (16 mg), **3** (16 mg), **4** (6 mg), **6** (15 mg), **7** (11 mg), **8** (11 mg), **9** (17 mg), **10** (8 mg), and **11** (23 mg). Fraction 3 was separated using MCI gel column chromatography and eluted with methanol–water (85–100%) to give 3 subfractions (fractions 3a–3c). Subfraction 3b (methanol/water, 90%) was purified using Sephadex LH-20 (acetone as eluent) columns to give compounds **5** (8 mg) and **12** (23 mg), and subfraction 3a (methanol/water, 85%) was further recrystallized to obtain 452 mg of compound **1**.

Leptolignan A (**7**), white solid. ${\left[\alpha \right]}_{D}^{25}$ = +138° (*c* = 0.1, MeOH). UV (MeOH): 296 (3.97), 230 (4.09). IR (KBr): 3442, 2955, 1632, 1484, 932 cm^−1^_._ HR-ESI-MS *m*/*z*: 499.1208 [M + Na]^+^_._ 1D NMR: Table 1 and Table 2. 

Leptolignan B (**8**), white solid. ${\left[\alpha \right]}_{D}^{25}$ = +16° (*c* = 0.1, MeOH). UV (MeOH): 296 (2.99), 230 (3.13). IR (KBr): 3440, 2895, 1736, 1629, 1485, 940 cm^−1^_._ HR-ESI-MS *m*/*z*: 511.1220 [M + Na]^+^. 1D NMR: Table 1 and Table 2.

Leptolignan C (**9**), white solid. ${\left[\alpha \right]}_{D}^{25}$ = +167° (*c* = 0.1, MeOH). UV (MeOH): 296 (3.80), 228 (4.08). IR (KBr): 3441, 2926, 1631, 1466, 933 cm^−1^_._ HR-ESI-MS *m*/*z*: 469.1113 [M + Na]^+^. 1D NMR: Table 1 and Table 2.

Leptolignan D (**10**), white solid. ${\left[\alpha \right]}_{D}^{25}$ = + 3° (*c* = 0.1, MeOH). UV (MeOH): 285 (2.22), 204 (3.34). IR (KBr): 3424, 2896, 1629, 1486, 1428, 933 cm^−1^. HR-ESI-MS *m*/*z*: 425.0837 [M + Na]^+^. 1D NMR: Table 1 and Table 2.

### 3.4. Antifeedant Assay

Petri dishes were lined with a piece of wet filter paper, and four round sections of cabbage leaf with a diameter of 1 cm were placed at the edges of the filter paper in a cross shape. In total, 10 μL of the test compound (dissolved in acetone) at the same concentration (1000, 500, 200, or 100 μg/mL in different experiments) was added to two opposite leaves, and a vehicle control (acetone) was added to the others. Commercial neem oil was used as a positive control. The Petri dishes were then air-dried for 30 min. Armyworms (*Mythimna separata*) was obtained from Henan Jiyuan Baiyun Industrial. Two *M. separata* 2nd-instar larvae were placed at the center of a prepared Petri dish and were allowed to feed. After 24 h, the consumption of leaves in each Petri dish was calculated according to a previously described method, and the EC_50_ (the effective dosage for 50% feeding reduction) values were used to evaluate the activity of each test compound [27,28].

### 3.5. Growth Inhibition Assay

Forage was purchased from Henan Jiyuan Baiyun Industrial. First, 1 g of compound **1** (dissolved in acetone) was added to 1 g of fresh forage to obtain final concentrations of 1, 0.5, 0.1, or 0 mg/g. The prepared forage was then used to feed a single 2nd-instar *M. separata* larva, with the final concentrations of 1, 0.5, 0.1, or 0 mg/each. Forage containing the same concentration of compound **1** was supplemented daily to ensure that the larva would not be stressed by starvation. The weight of each *M. separata* larva was measured and recorded every day, and the growth inhibition activity of compound **1** against *M. separata* was assessed using a previously described method [24]. Each group comprised five parallel experiments.

### 3.6. Determination of Nutritional Indexes

The nutritional indexes of 3rd-instar *M. separata* larvae, which had been starved for one day, were measured and calculated according to a previously published method [29]. Briefly, 300 μL of compound **1** (dissolved in acetone) was added to 1 g fresh maize leaves (B73, two-week-old seedlings) to reach a concentration of 1 mg/g. The treated leaves were weighed and then fed to 20 3rd-instar *M. separata* larvae. Following 72 h treatment, the weights of the leaves and larvae in each group were measured and the following nutritional indexes were calculated immediately: relative consumption rate (RCR), relative growth rate (RGR), larval weight gain (LWG), approximate digestibility (AD), assimilation rate (AR), efficiency of conversion of ingested food to body substance (ECI), and efficiency of conversion of digested food into growth (ECD).

### 3.7. Quantitative Analysis of Hormones through UPLC-MS/MS

Standard chemicals including ecdysone (E), and 20-hydroxyecdysone (20E), 2 deoxyecdysone (2DE), ketodiol (KD), and ketotriol (KT) were obtained from Sigma-Aldrich. *M. separata* larvae were allowed to feed on compound **1**-treated maize leaves for three days. Larvae were then harvested and 0.5 g of larval sample was added into 8 mL solution (including 75% methanol, 20% water, and 5% formic acid) for 90 min. The extracts were then desalted using HLB solid-phase extraction columns (Shimadzu GL InertSep HLB) and were quantitatively analyzed on a UPLC-MS/MS (Shimadzu 8050) equipped with a Shim-pack GIST-C_18_ column (2 μm, 2.1 × 50 mm). All of the samples were analyzed using the MRM mode following previous reports. The gradient elution program used 0.1% formic acid water as solvent A and acetonitrile as solvent B, and was set as follows: the solvent gradient was linearly changed from 5 to 95% solvent B over the first 12 min, then maintained at 95% solvent B for 2 min, reduced from 95 to 5% B over 4 min, and finally held at 5% B for 5 min [30].

## 4. Conclusions

Plant origin is an important factor in the diversity of plant metabolites [31]. In this study, four new phrymarolin-type lignans, leptolignans A–D (**7**–**10**), along with eight known lignans, were isolated from whole *P. leptostachya* plants grown in Shenyang City. Of these, leptolignan A showed the most significant antifeedant activity against *M*. *separata* in a dual-choice bioassay. Further mechanism analysis showed that lignans were able to reduce the growth and nutritional indexes in *M*. *separata* larvae and that these biological activities might result from decreases in the accumulation of ecdysone and 20-hydroxyecdysone. These findings provide more information on the structural diversity of lignans from *P. leptostachya* and suggest a potential mechanism by which lignans may confer resistance against *M*. *separata*.

## Figures and Tables

**Table 1:** ^1^H NMR [600 MHz, *δ*_H_, mult. (*J* in Hz)] spectral data for compounds **7–10** in acetone-*d*_6_.

Position	7	8	9	10
1	-	-	-	-
2	-	6.61 (1H, d, 2.4)	-	6.58 (1H, d, 2.3)
3	-	-	-	-
4	-	-	-	-
5	6.33 (1H, s)	6.73 (1H, d, 8.4)	6.51 (1H, d, 8.4)	6.61 (1H, dd, 2.2, 8.4)
6	-	6.52 (1H, dd, 2.4, 8.4)	6.68 (1H, d, 8.4)	6.45 (1H, dd, 2.3, 8.4)
7	5.11 (1H, s)	5.67 (1H, s)	5.30 (1H, s)	5.33 (1H, s)
8	-	-	-	-
9a	4.07 (1H, d, 11.5)	4.44 (1H, d, 11.0)	4.25 (1H, d, 9.6)	4.06 (1H, d, 9.7)
9b	3.48 (1H, d, 11.5)	3.63 (1H, overlap)	3.68 (1H, dd, 1.5, 9.6)	3.78 (1H, overlap)
10	5.79 (2H, m)	5.95 (2H, overlap)	5.99 (2H, s)	5.81 (2H, overlap)
1′	-	-	-	-
2′	6.95 (1H, s)	-	7.09 (1H, s)	-
3′	-	-	-	-
4′	-	-	-	-
5′	6.55 (1H, s)	6.45 (1H, s)	6.70 (1H, s)	6.30 (1H, dd, 8.3,14.2)
6′	-	-	-	6.75 (1H, d, 8.1)
7′	4.69 (1H, d, 6.1)	4.96 (1H, d, 8.1)	4.86 (1H, d, 6.1)	5.22 (1H, s)
8′	2.34 (1H, dd, 3.9, 9.5)	3.46 (1H, m)	2.51 (1H, m)	2.99 (1H, m)
9′a	4.44 (1H, dd, 7.6, 8.8)	4.14 (1H, dd, 6.2,9.2)	4.43 (1H, dd, 7.4, 9.1)	3.78 (1H, overlap)
9′b	3.81 (1H, d,2.4)	3.67 (1H, overlap)	4.03 (1H, dd, 2.4, 9.1)	3.40 (1H, m)
10′	5.81 (2H, m)	5.95 (2H, overlap)	5.96 (2H, d, 2.5)	5.81 (2H, overlap)
6′-OMe	3.68 (3H, s)	3.78 (3H, s)	3.83 (3H, s)	
2-OMe	3.82 (3H, s)		3.96 (3H, s)	
6-OMe	3.65 (3H, s)			
2′-OMe		3.96 (3H, s)		
8-OAC		2.16 (3H, s)		

**Table 2:** ^13^C NMR (150 MHz, *δ*_C_) spectral data for compounds **7–10** in acetone-*d*_6_.

Position	7	8	9	10
1	132.2, s	153.1, s	144.7, s	153.0, s
2	139.2, s	101.5, d	137.2, s	101.1, d
3	132.4, s	144.0, s	139.0, s	143.6, s
4	145.5, s	149.0, s	145.8, s	148.9, s
5	91.1, d	108.6, d	102.5, d	108.6, d
6	149.0, s	110.9, d	113.4, d	110.3, d
7	105.7, d	104.3, d	105.4, d	106.2, d
8	93.4, s	97.7, s	93.1, s	90.3, s
9	78.5, t	76.3, t	78.5, t	78.0, t
10	102.0, t	102.2, t	102.4, t	102.2, t
1′	124.0, s	112.2, s	123.9, s	122.1, s
2′	106.7, d	144.1, s	106.7, d	138.4, s
3′	142.0, s	132.5, s	142.0, s	135.3, s
4′	148.0, s	150.6, s	148.0, s	148.8, s
5′	95.2, d	90.8, d	95.2, d	100.8, d
6′	152.1, s	155.2, s	152.1, s	120.2, d
7′	85.0, d	81.1, d	85.0, d	80.5, d
8′	59.5, d	53.9, d	59.4, d	52.5, d
9′	71.8, t	68.8, t	71.5, t	67.8, t
10′	102.0, t	102.1, t	102.0, t	102.0, t
6′-OMe	57.4, q	57.2, q	56.6, q	
2-OMe	60.4, q		60.5, q	
6-OMe	56.7, q			
2′-OMe		60.4, q		
8-OAC		21.1, q		
		171.3, s		

## Data Availability

Data are contained within the article and Supplementary Materials.

## Supplementary Materials

The following supporting information can be downloaded at: https://www.mdpi.com/article/10.3390/ijms25137081/s1.
