# Supplementary material for: Four New Furofuran Lignans from Phryma leptostachya Inhibit the Accumulation of Molting Hormones in Armyworm
Source: Int J Mol Sci. 2024 Jun 27;25(13):7081. doi: 10.3390/ijms25137081 (PMC11240949; doi:10.3390/ijms25137081)
Supplement: Supplementary file 1 [file ijms-25-07081-s001.zip › ijms-3034022-supplementary.pdf]

Four new furofuran lignans from *Phryma leptostachya* inhibit the accumulation of molting hormones in armyworm

Supporting Information

Jiaming Zhang†, Qi Cong†, Yuyao Sun, Juan Hua*, Shihong Luo*

Engineering Research Center of Protection and Utilization of Plant Resources, College of Bioscience and Biotechnology, Shenyang Agricultural University, Shenyang, 110866, Liaoning Province, China

†These authors contributed equally to this manuscript.

*Corresponding Authors

Juan Hua, huajuan@syau.edu.cn; Prof. Shihong Luo, E-mail address: luoshihong@syau.edu.cn (http://orcid.org/0000-0003-3500-3466)

Table of Contents

Table S1. ^{13}C NMR (150 MHz, δ_C) spectral data for compounds **1**,**3**,**11**, and **12** in acetone-d_6**Error! Bookmark not defined.**

Figure S1. ^{1}H NMR spectrum of leptolignan A (**7**) recorded at 600 MHz in acetone-d_6**Error! Bookmark not defined.**

Figure S2. ^{13}C NMR and DEPT spectra of leptolignan A (**7**) recorded at 150 MHz in acetone-d_6......S6

Figure S3. HSQC spectrum of leptolignan A (**7**) in acetone-$d_6$2

Figure S4. HMBC spectrum of leptolignan A (**7**) in acetone-d_6......S8

Figure S5. ^{1}H−^{1}H COSY spectrum of leptolignan A (**7**) in acetone-d_6......S9

Figure S6. ^{1}H−^{1}H ROESY spectrum of leptolignan A (**7**) in acetone-d_6......S10

Figure S7. HR-ESI-MS spectrum of leptolignan A (**7**) S11

Figure S8. IR spectrum of leptolignan A (**7**) S12

Figure S9. CD spectrum of leptolignan A (**7**)......S13

Figure S10. Optical rotation data of leptolignan A (**7**) S14

Figure S11. ^{1}H NMR spectrum of leptolignan B (**8**) recorded at 600 MHz in acetone-d_6......S15

Figure S12. ^{13}C NMR and DEPT spectra of leptolignan B (**8**) recorded at 150 MHz in acetone-d_6......S16

Figure S13. HSQC spectrum of leptolignan B (**8**) in acetone-d_6......S17

Figure S14. HMBC spectrum of leptolignan B (**8**) in acetone-d_6......S18

Figure S15. ^{1}H−^{1}H COSY spectrum of leptolignan B (**8**) in acetone-d_6......S19

Figure S16. ^{1}H−^{1}H ROESY spectrum of leptolignan B (**8**) in acetone-d_6......S20

Figure S17. HR-ESI-MS spectrum of leptolignan B (**8**) S21

Figure S18. IR spectrum of leptolignan B (**8**)......S22

Figure S19. CD spectrum of leptolignan B (**8**) S23

Figure S20. Optical rotation data of leptolignan B (**8**) S24

Figure S21. ^{1}H NMR spectrum of leptolignan C (**9**) recorded at 600 MHz in acetone-d_6......S25

Figure S22. ^{13}C NMR and DEPT spectra of leptolignan C (**9**) recorded at 150 MHz in acetone-d_6......S26

Figure S23. HSQC spectrum of leptolignan C (**9**) in acetone-d_6......S27

Figure S24. HMBC spectrum of leptolignan C (**9**) in acetone-d_6......S28

Figure S25. ^{1}H−^{1}H COSY spectrum of leptolignan C (**9**) in acetone-d_6......S29

Figure S26. ^{1}H−^{1}H ROESY spectrum of leptolignan C (**9**) in acetone-d_6......S30

Figure S27. HR-ESI-MS spectrum of leptolignan C (**9**) S31

Figure S28. IR spectrum of leptolignan C (**9**)......S32

Figure S29. CD spectrum of leptolignan C (**9**) S33

Figure S30. Optical rotation data of leptolignan C (**9**) S34

Figure S31. ^{1}H NMR spectrum of leptolignan D (**10**) recorded at 600 MHz in acetone-d_6......S35

Figure S32. ^{13}C NMR and DEPT spectra of leptolignan D (**10**) recorded at 150 MHz in acetone-d_6......S36

Figure S33. HSQC spectrum of leptolignan D (**10**) in acetone-d_6......S37

Figure S34. HMBC spectrum of leptolignan D (**10**) in acetone-d_6..........................S38
Figure S35. ^{1}H−^{1}H COSY spectrum of leptolignan D (**10**) in acetone-d_6...............S39
Figure S36. ^{1}H−^{1}H ROESY spectrum of leptolignan D (**10**) in acetone-d_6.............S40
Figure S37. HR-ESI-MS spectrum of leptolignan D (**10**) S41
Figure S38. IR spectrum of leptolignan D (**10**) S42
Figure S39. CD spectrum of leptolignan D (**10**)..S43
Figure S40. Optical rotation data of leptolignan D (**10**) S44

Table S1. ^{13}C NMR (150 MHz, δ_C) spectral data for compounds **1**,**3**,**11**, and **12** in acetone-d_6.

Position	**1**	**3**	**11**	**12**
1	113.3, s	113.0, s	120.4, s	152.1, s
2	155.0, s	152.3, s	124.0, d	101.2, d
3	132.4, s	142.0, s	148.9, s,	148.0, s
4	150.2, s	150.1, s	143.7, s	143.7, s
5	90.7, d	95.4, d	108.6, d	108.6, d
6	143.8, s	106.4, d	110.3, d	110.3, d
7	79.2, d	78.1, d	103.2, d	103.5, d
8	98.3, s	98.5, s	93.0, s	92.9, s
9	76.8, t	73.0, t	78.6, t	78.5, t
10	102.0, t	102.0, t	102.0, t	102.2, t
1'	114.2, s	131.9, s	120.4, s	123.9, s
2'	154.9, s	143.7, s	139.3, s	106.7, d
3'	132.1, s	123.9, s	135.5, s	142.0, s
4'	150.0, s	148.1, s	149.1, s	149.0, s
5'	90.6, d	90.4, d	100.9, d	95.2, d
6'	143.6, s	155.1, s	101.1, d	153.0, s
7'	79.2, d	82.6, d	87.4, d	85.0, d
8'	58.3, d	61.9, d	59.1, d	59.6, d
9'	71.8, t	71.1, t	70.7, t	71.2, t
10'	101.9, t	101.9, t	102.2, t	102.0, t
6'-OMe	60.3, q	56.9, q	-	56.6, q
2-OMe	57.2, q	-	-	
6-OMe	60.0, q	59.9, q	-	
2'-OMe	57.0, q	56.7, q	-	
8-OAC	21.0, q	20.9, q	-	
	169.8, s	170.0, s	-	

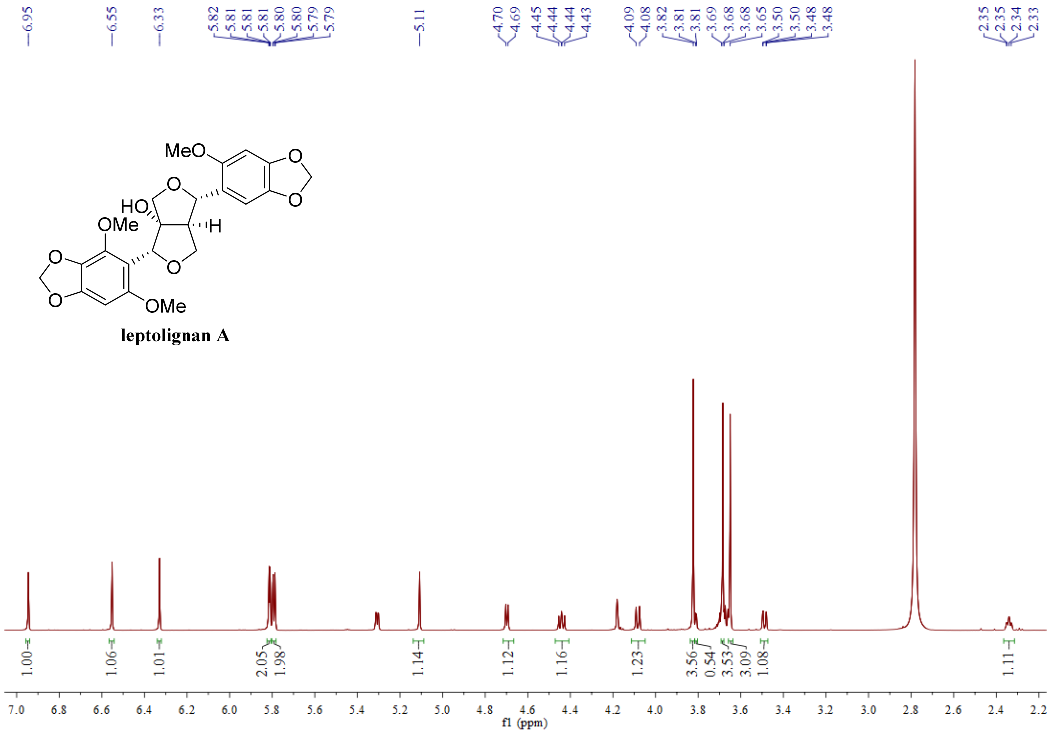

Figure S1. ^{1}H NMR spectrum of leptolignan A (**7**) recorded at 600 MHz in acetone-d_6.

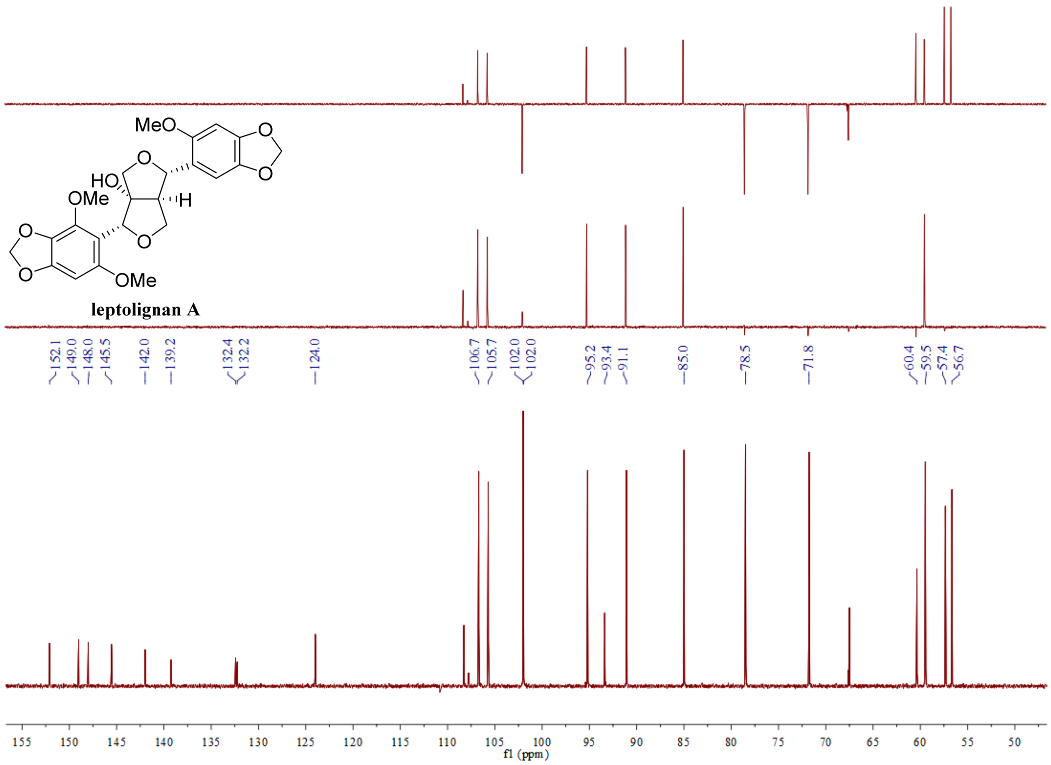

Figure S2. ^{13}C NMR and DEPT spectra of leptolignan A (**7**) recorded at 150 MHz in acetone-*d*6.

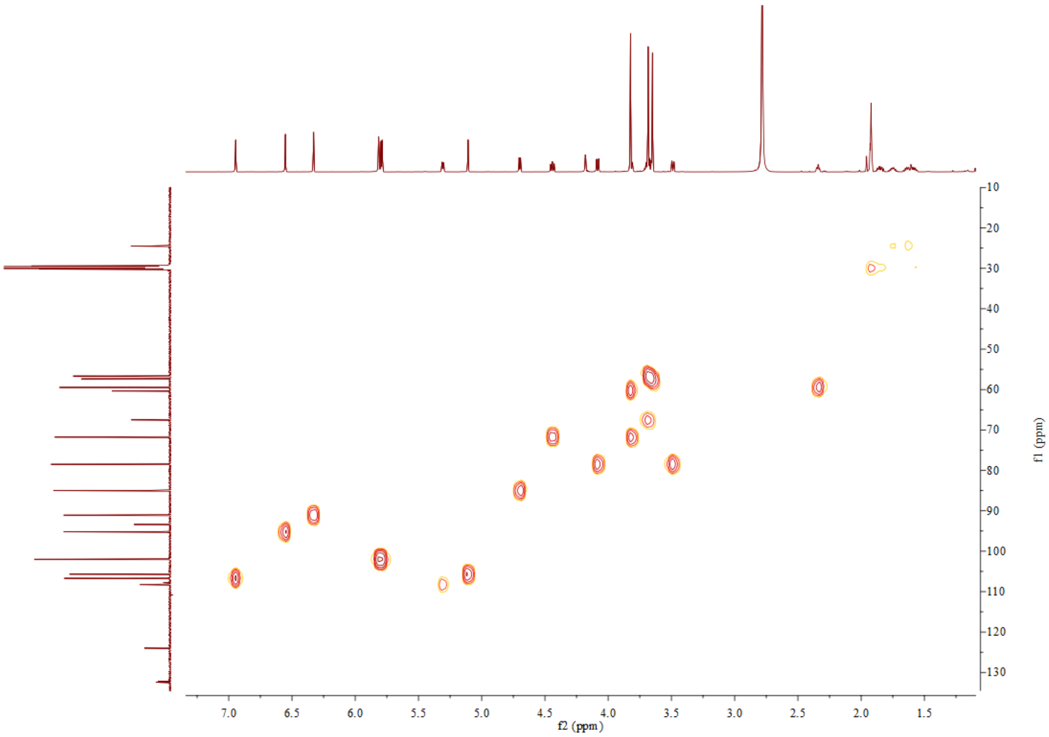

Figure S3. HSQC spectrum of leptolignan A (**7**) in acetone-d_6.

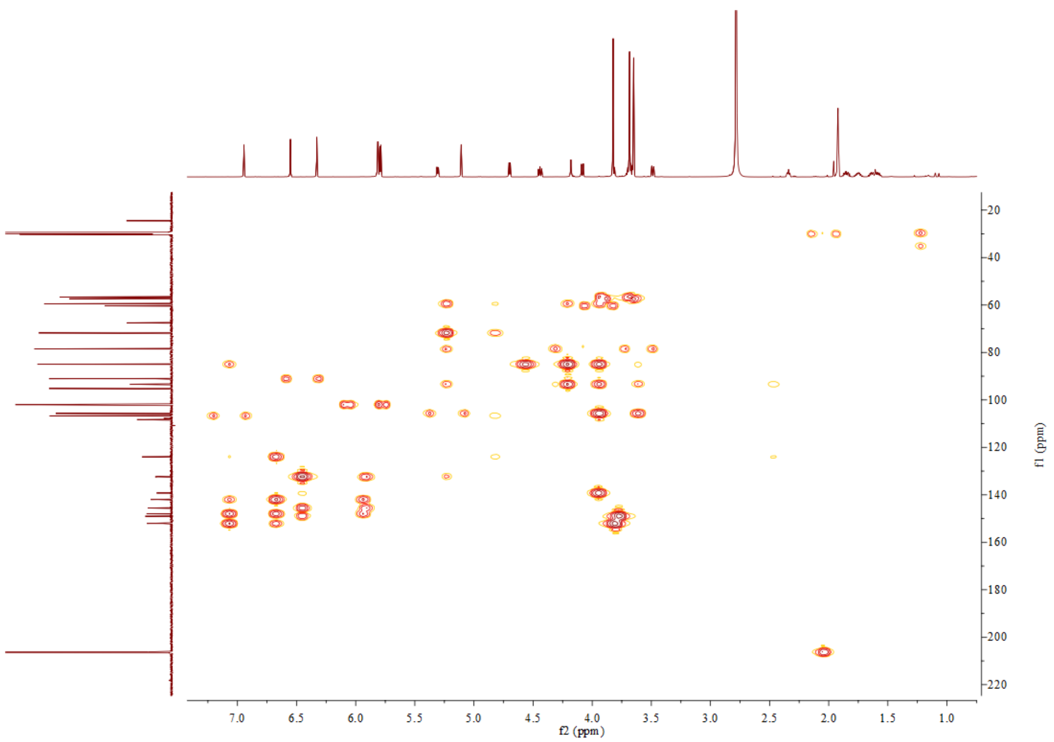

Figure S4. HMBC spectrum of leptolignan A (**7**) in acetone-*d*6.

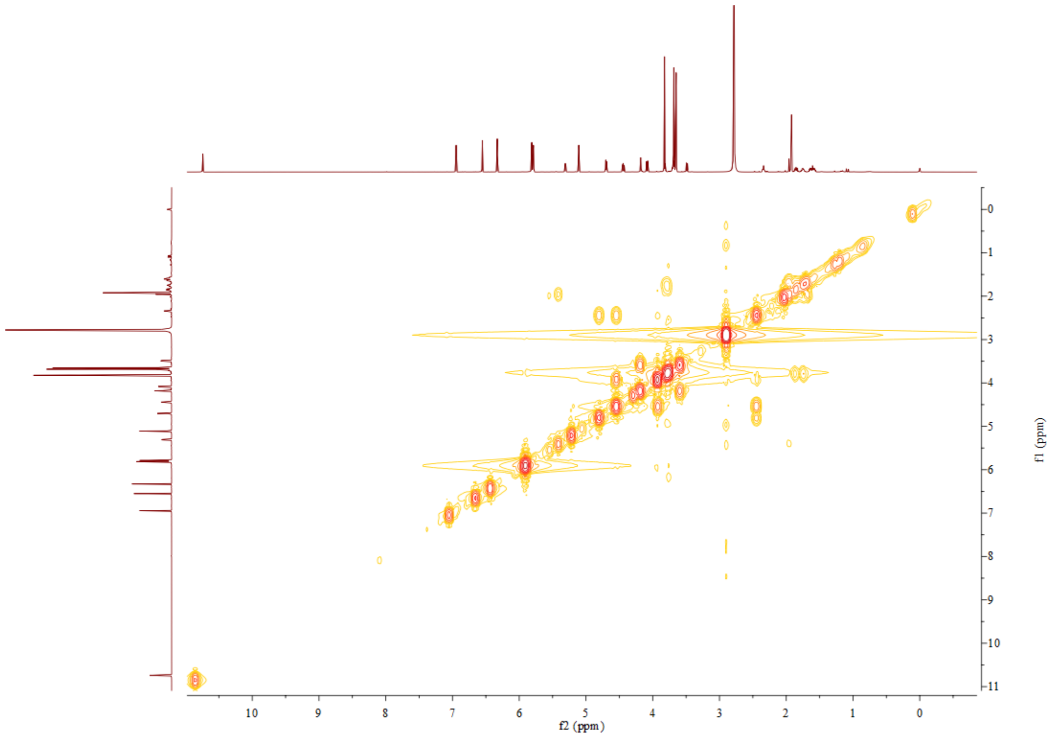

Figure S5. ^{1}H–^{1}H COSY spectrum of leptolignan A (**7**) in acetone-d_6.

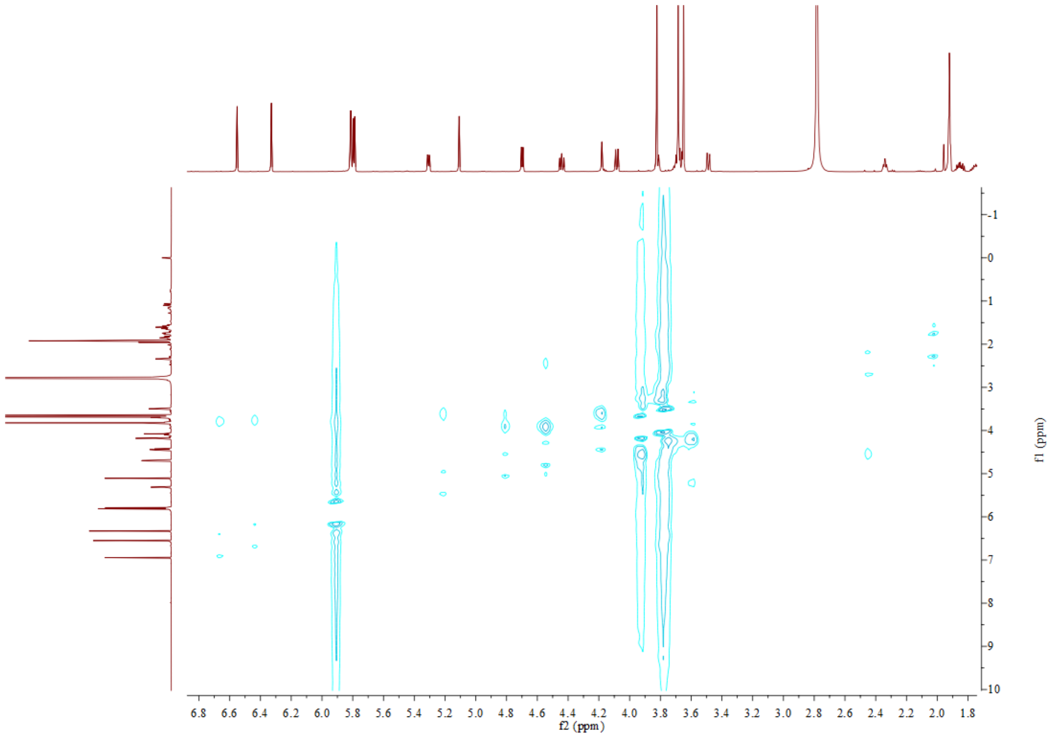

Figure S6. $^{1}H-^{1}H$ ROESY spectrum of leptolignan A (**7**) in acetone-d_6.

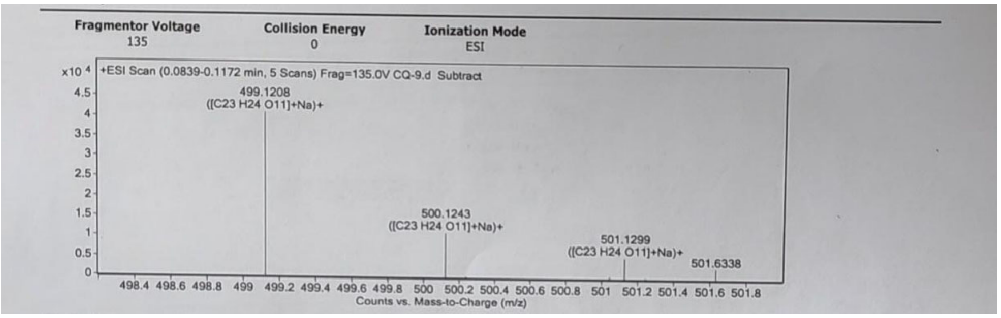

Figure S7. HR-ESI-MS spectrum of leptolignan A (**7**).

Formula	CalculatedMass	CalculatedMz	Mz	Diff. (mDa)	Diff. (ppm)	DBE
$C_{23}H_{24}O_{11}$	476.1319	499.1211	499.1208	0.30	0.60	12.0000

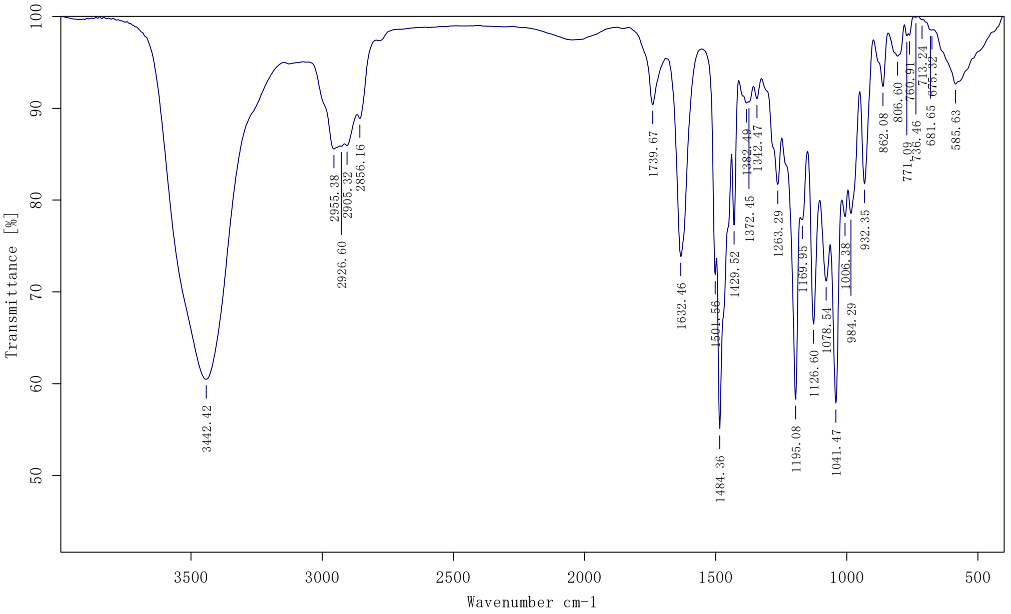

Sample Name: CQ-9
Sample Form: KBr
Path of File: E:\data
Date of Measurement: 2024/5/15
Resolution: 4
Aperture Setting: 6 mm
Number of Background Scans: 16
Number of Sample Scans: 16
Beamsplitter Setting: KBr
Source Setting: MIR
Instrument Type: BRUKER VERTEX 70
Soft Version: OPUS8.1

Figure S8. IR spectrum of leptolignan A (**7**).

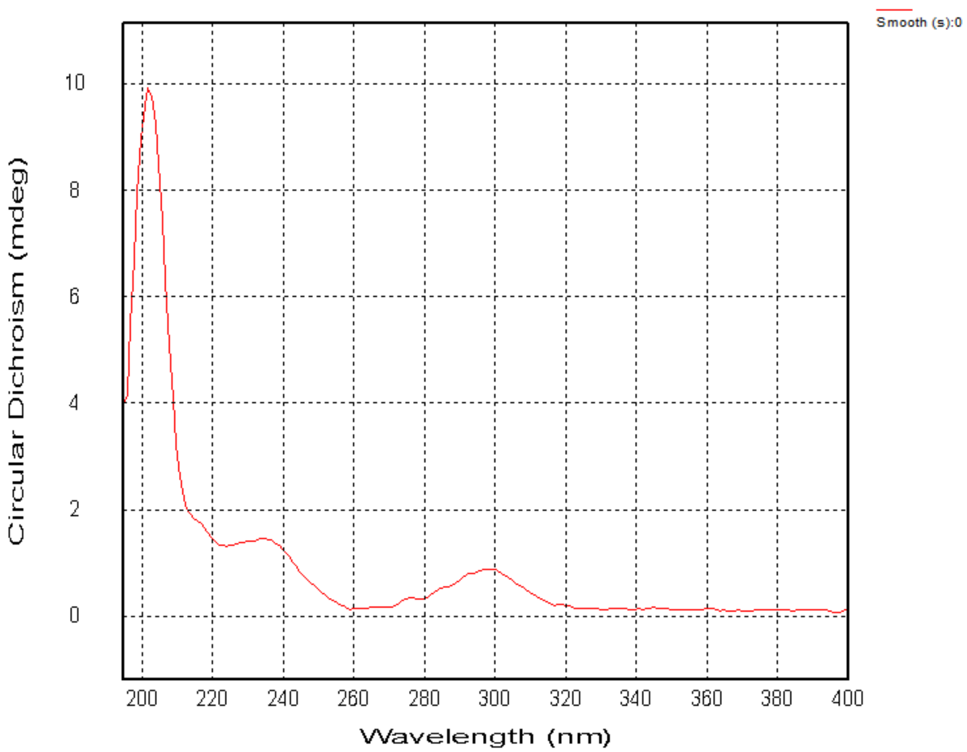

Figure S9. CD spectrum of leptolignan A (**7**).

n	Average	Std.Dev.	% RSD	Maximum	Minimum
5	138.40	0.55	0.39	139.00	138.00

S.No	Sample ID	Time	Result	Scale	OR °Arc	WLG.nm	Lg.mm	Conc.g/100ml	Temp.
1	CQ-9	11:25:38 AM	138.00	SR	0.138	589	100.00	0.100	25.0
2	CQ-9	11:25:44 AM	138.00	SR	0.138	589	100.00	0.100	25.0
3	CQ-9	11:25:50 AM	138.00	SR	0.138	589	100.00	0.100	25.0
4	CQ-9	11:25:57 AM	139.00	SR	0.139	589	100.00	0.100	25.0
5	CQ-9	11:26:03 AM	139.00	SR	0.139	589	100.00	0.100	25.0

Figure S10. Optical rotation data of leptolignan A (**7**).

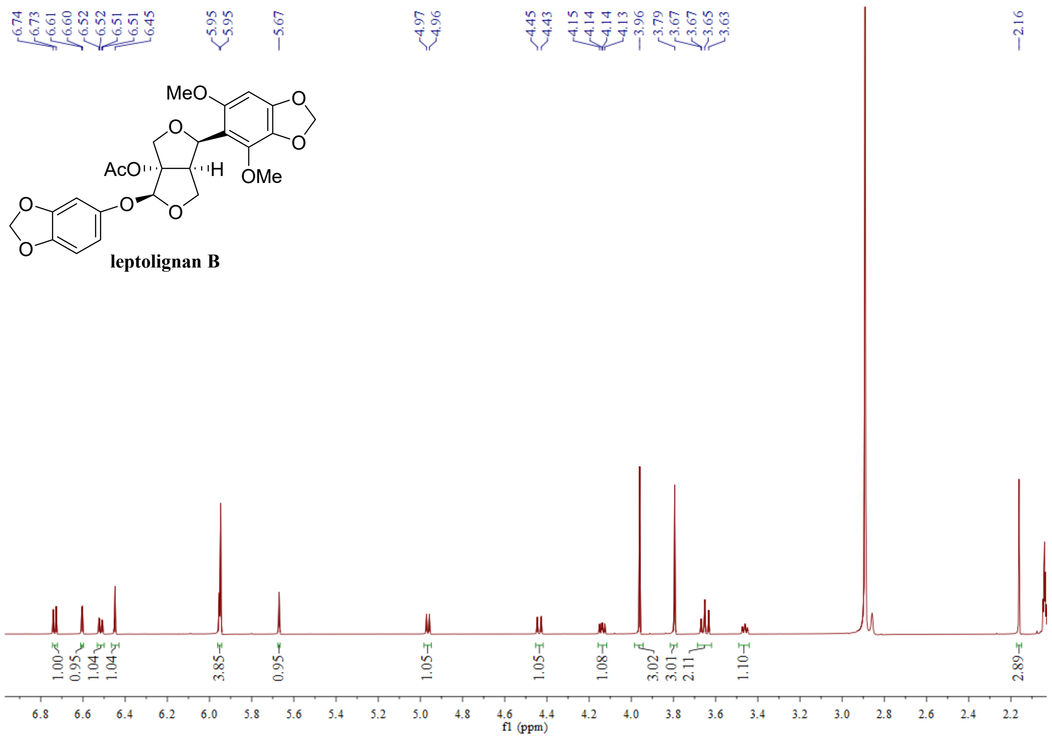

Figure S11. ^{1}H NMR spectrum of leptolignan B (**8**) recorded at 600 MHz in acetone-d_6.

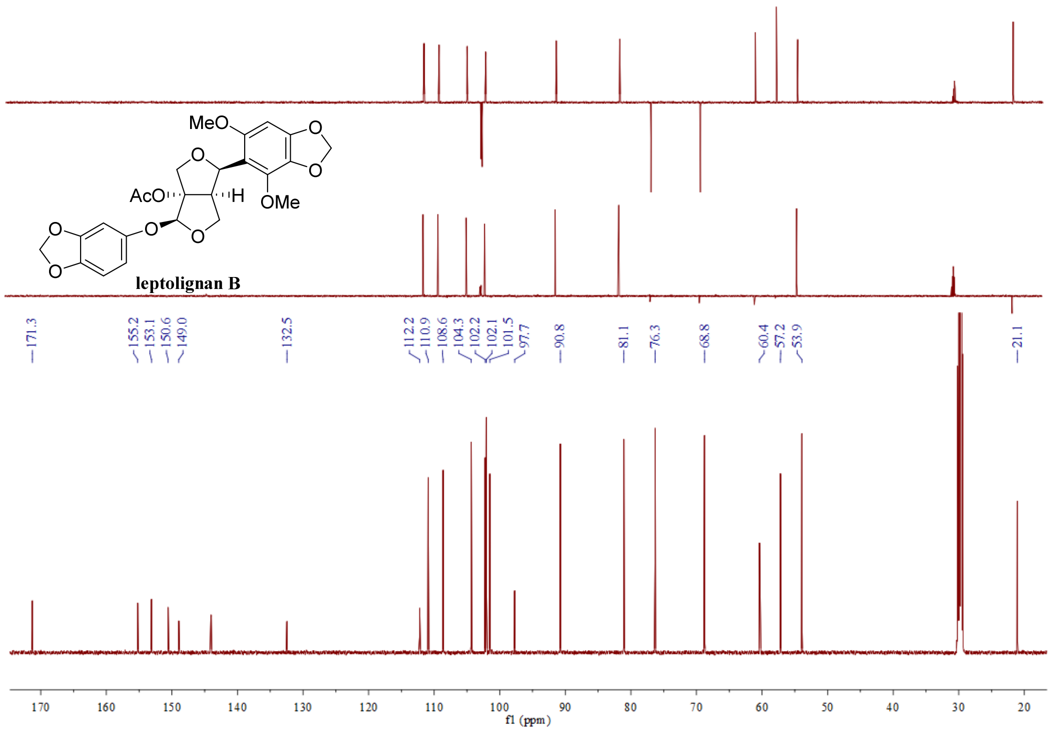

Figure S12. ^{13}C NMR and DEPT spectra of leptolignan B (**8**) recorded at 150 MHz in acetone-d_6.

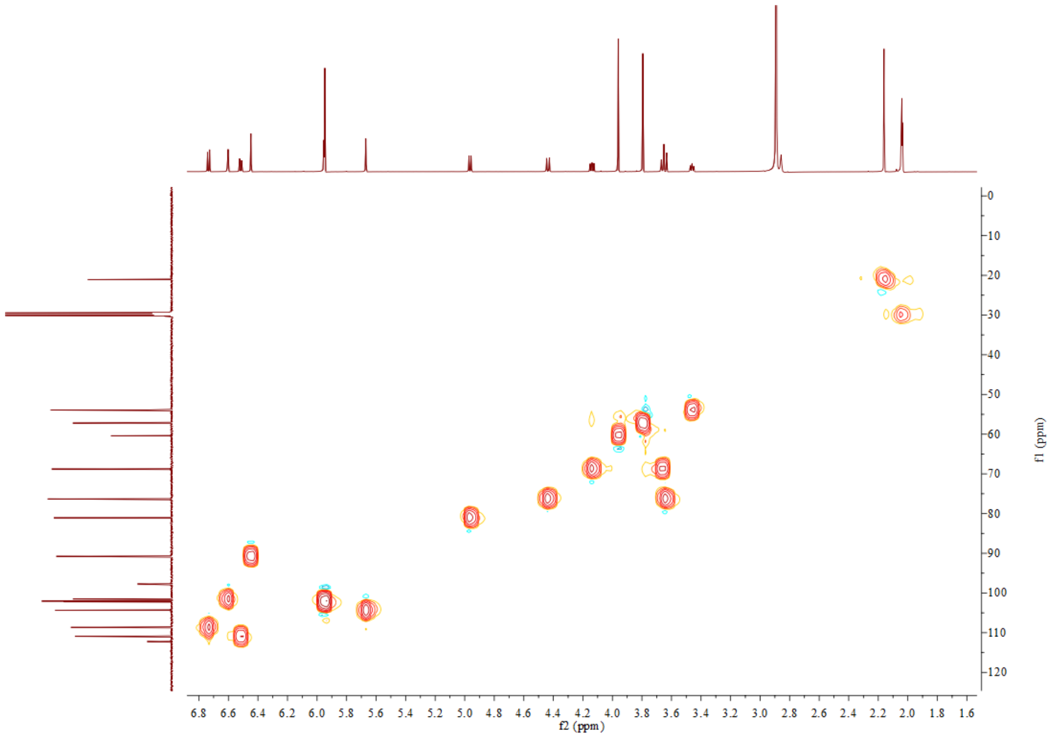

Figure S13. HSQC spectrum of leptolignan B (**8**) in acetone-d_6.

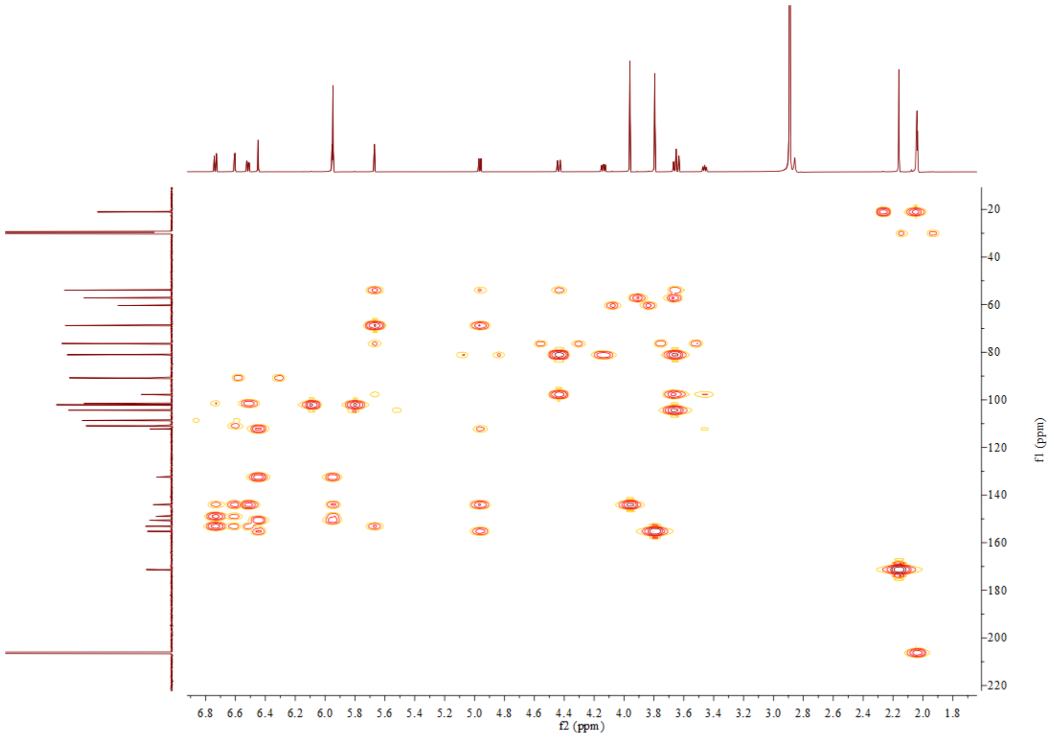

Figure S14. HMBC spectrum of leptolignan B (**8**) in acetone-d_6.

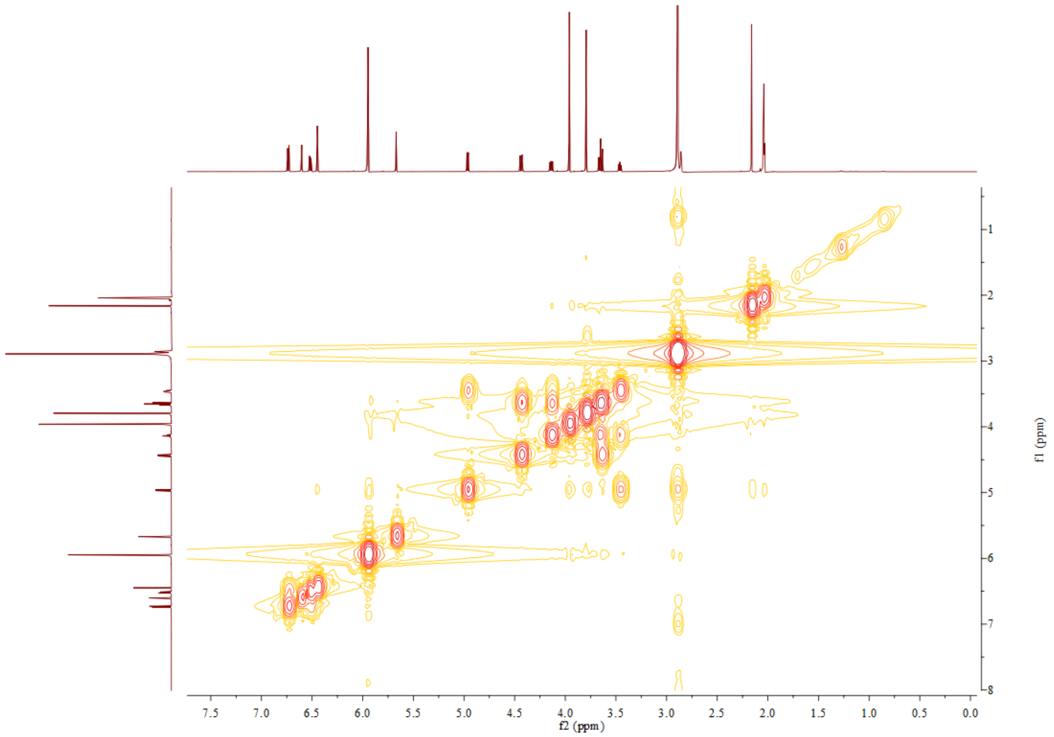

Figure S15. ¹H−¹H COSY spectrum of leptolignan B (**8**) in acetone-d_6.

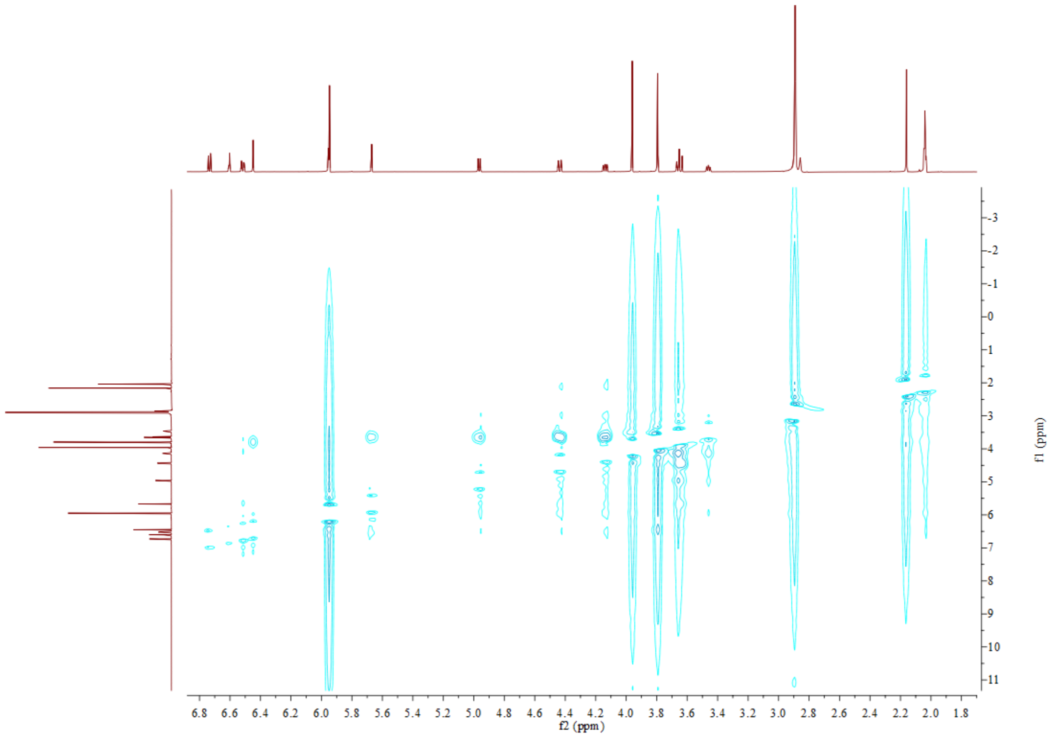

Figure S16. ^{1}H–^{1}H ROESY spectrum of leptolignan B (**8**) in acetone-d_6.

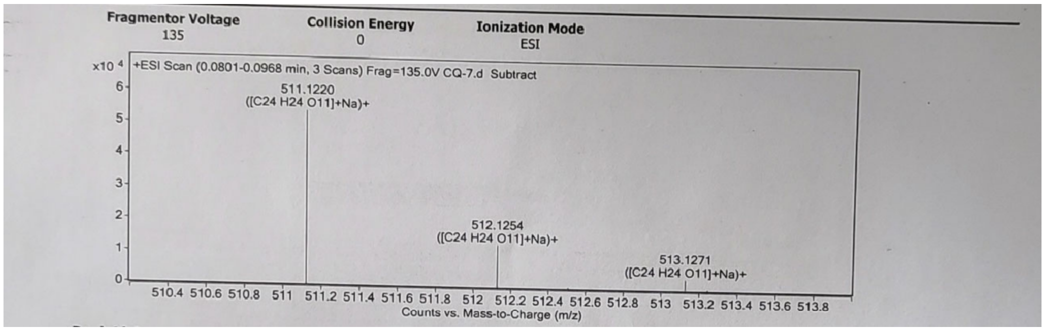

Figure S17. HR-ESI-MS spectrum of leptolignan B (**8**).

Formula	CalculatedMass	CalculatedMz	Mz	Diff. (mDa)	Diff. (ppm)	DBE
$C_{24}H_{24}O_{11}$	488.1319	511.111	511.1220	-0.90	-1.76	13.0000

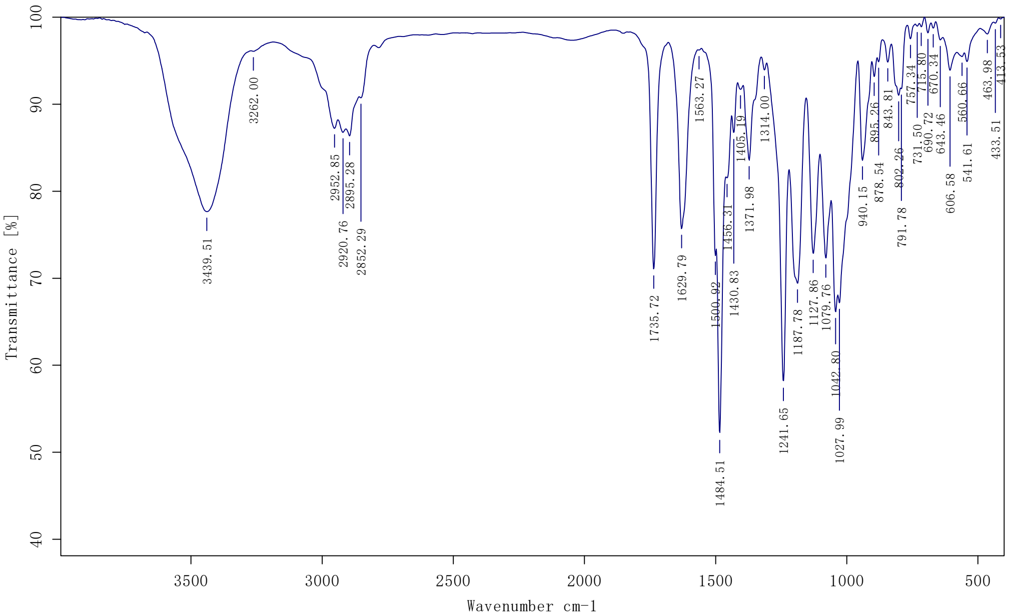

Sample Name: CQ-7	Resolution: 4	Beamsplitter Setting: KBr
Sample Form: KBr	Aperture Setting: 6 mm	Source Setting: MIR
Path of File: E:\data	Number of Background Scans: 16	Instrument Type: BRUKER VERTEX 70
Date of Measurement: 2024/5/15	Number of Sample Scans: 16	Soft Version: OPUS8.1

Figure S18. IR spectrum of leptolignan B (**8**).

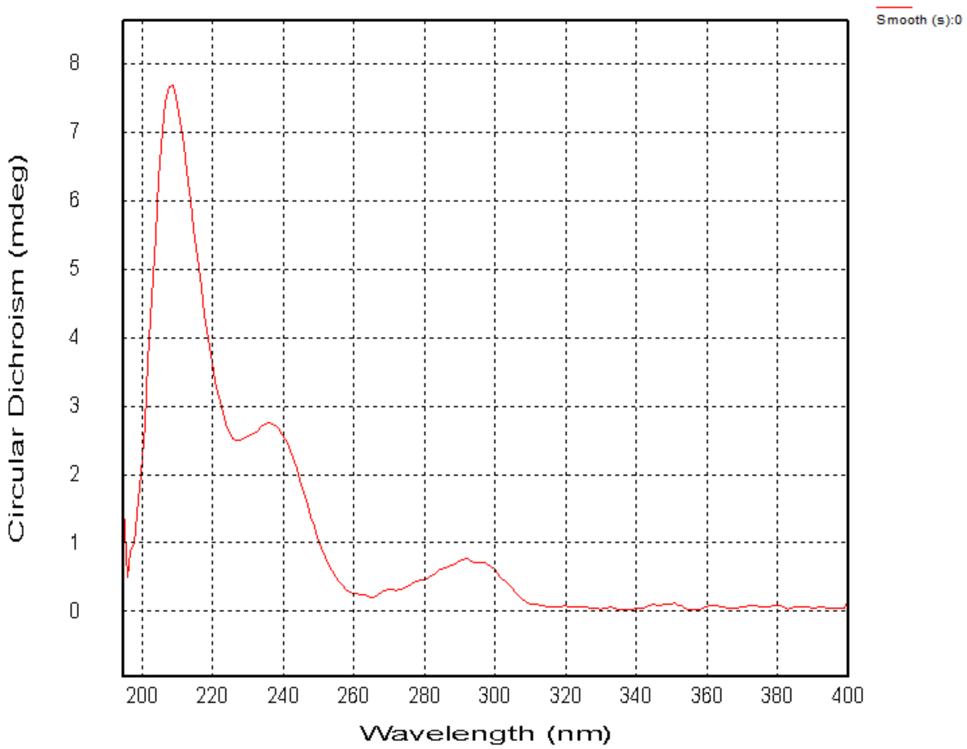

Figure S19. CD spectrum of leptolignan B (**8**).

n	Average	Std.Dev.	% RSD	Maximum	Minimum
5	15.85	0.08	0.50	15.89	15.71

S.No	Sample ID	Time	Result	Scale	OR °Arc	WLG.nm	Lg.mm	Conc.g/100ml	Temp.
1	CQ-7	11:14:45 AM	15.89	SR	0.089	589	100.00	0.560	25.0
2	CQ-7	11:14:51 AM	15.71	SR	0.088	589	100.00	0.560	25.0
3	CQ-7	11:14:57 AM	15.89	SR	0.089	589	100.00	0.560	25.0
4	CQ-7	11:15:04 AM	15.89	SR	0.089	589	100.00	0.560	25.0
5	CQ-7	11:15:10 AM	15.89	SR	0.089	589	100.00	0.560	25.0

Figure S20. Optical rotation data of leptolignan B (**8**).

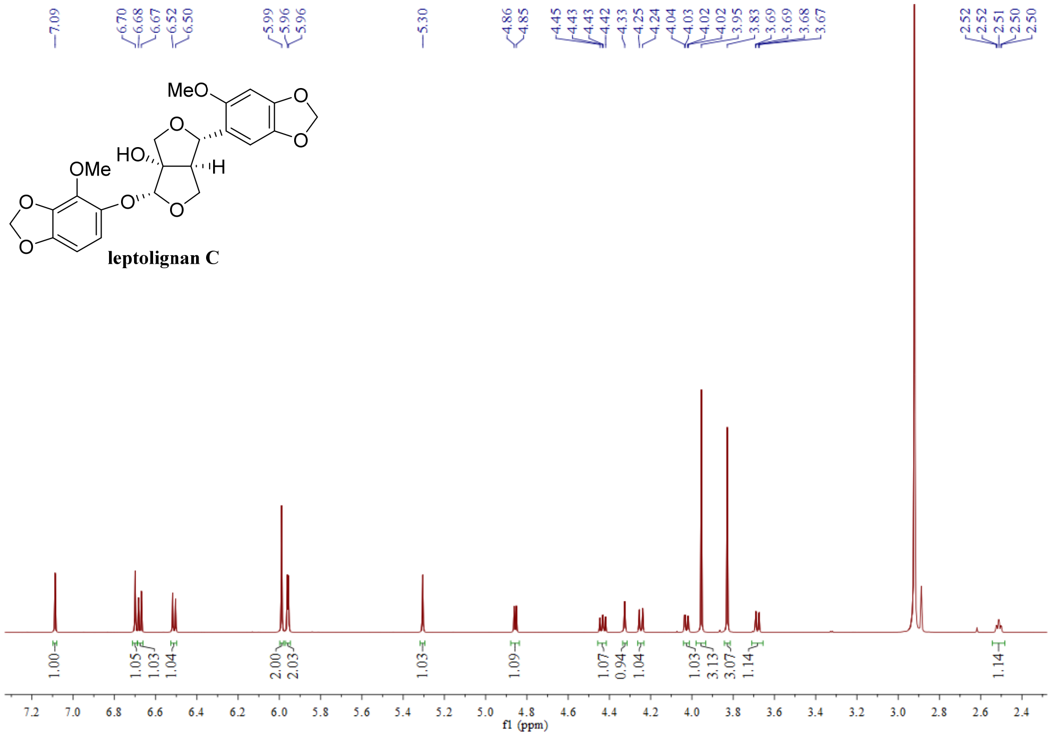

Figure S21. ^{1}H NMR spectrum of leptolignan C (**9**) recorded at 600 MHz in acetone-d_6.

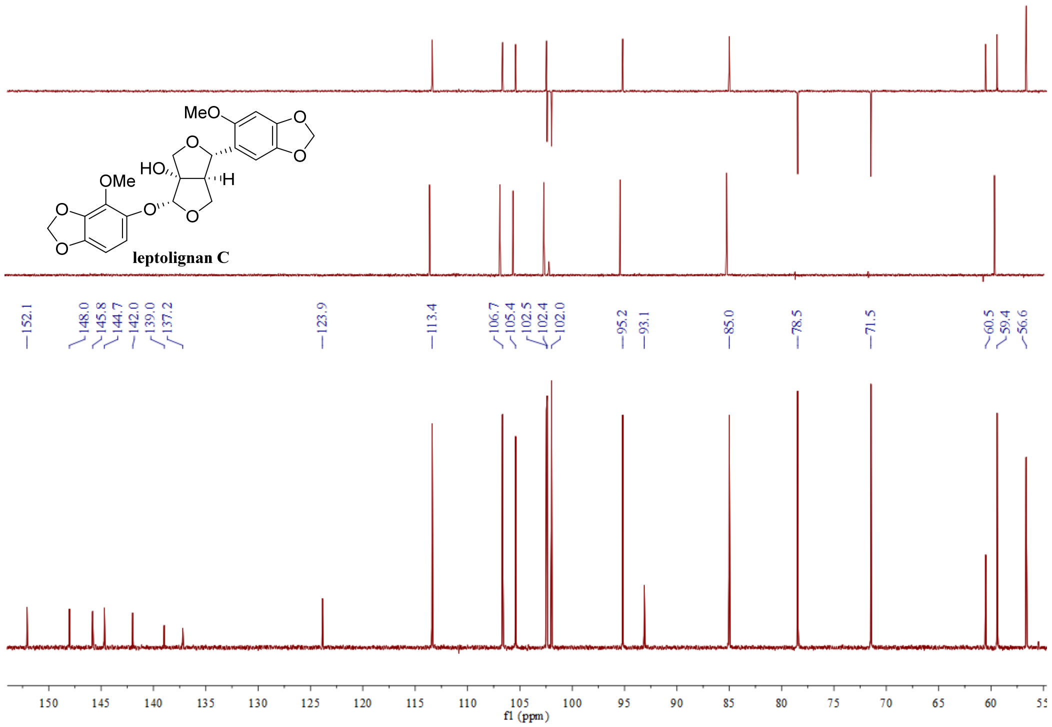

Figure S22. ^{13}C NMR and DEPT spectra of leptolignan C (**9**) recorded at 150 MHz in acetone-d_6.

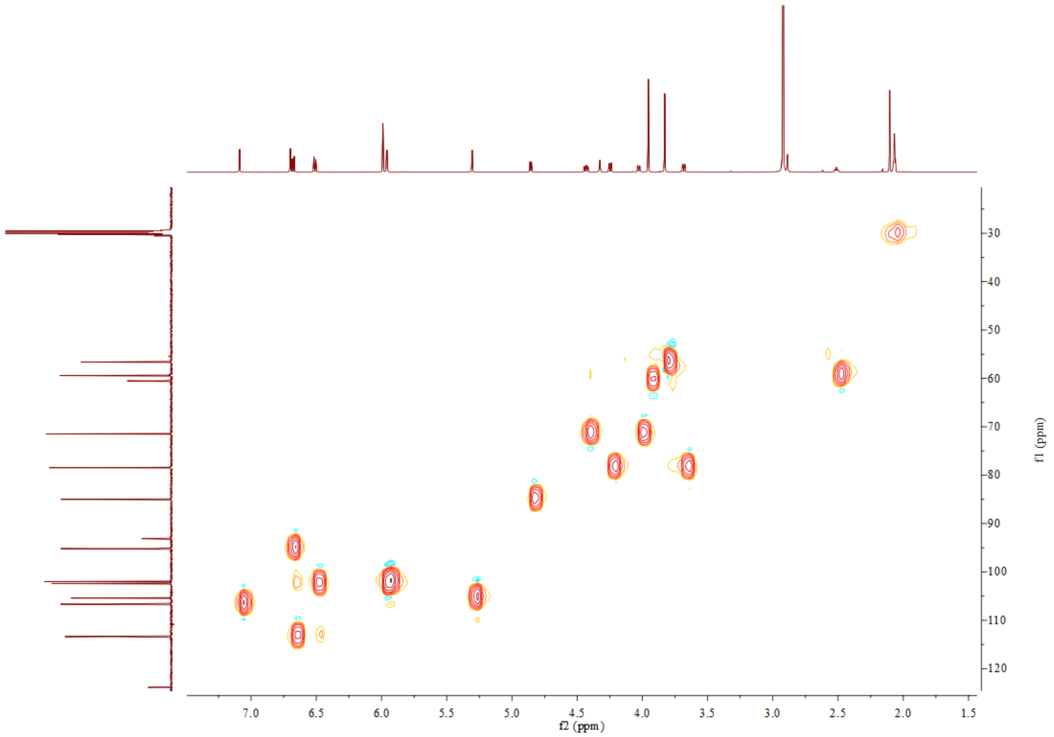

Figure S23. HSQC spectrum of leptolignan C (**9**) in acetone-d_6.

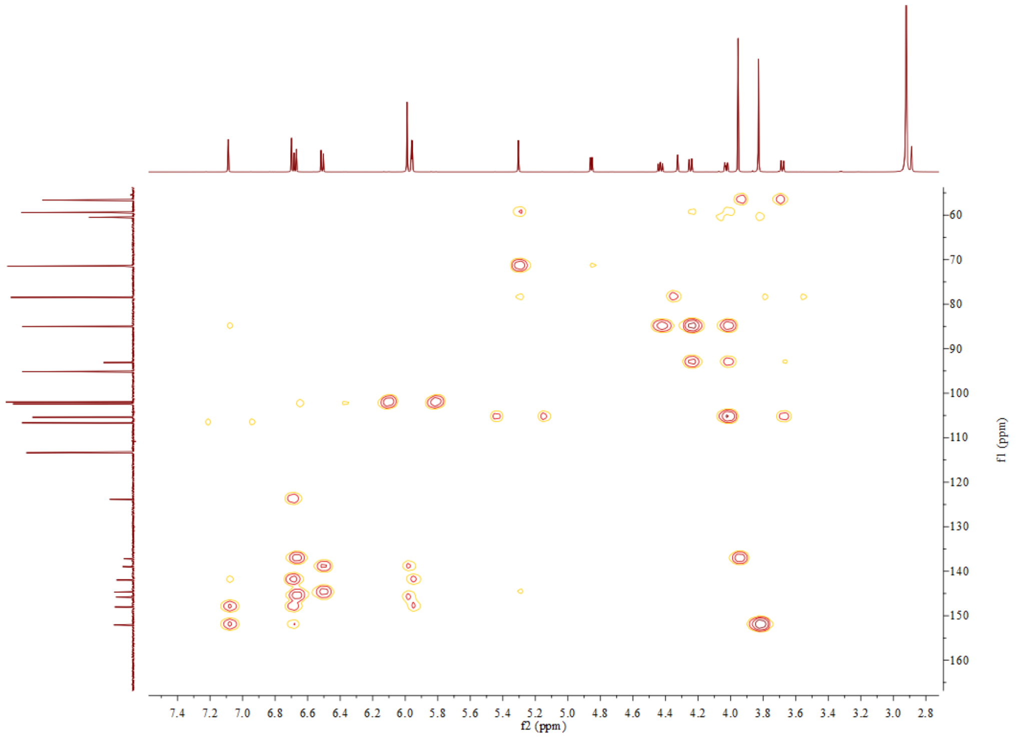

Figure S24. HMBC spectrum of leptolignan C (**9**) in acetone-d_6.

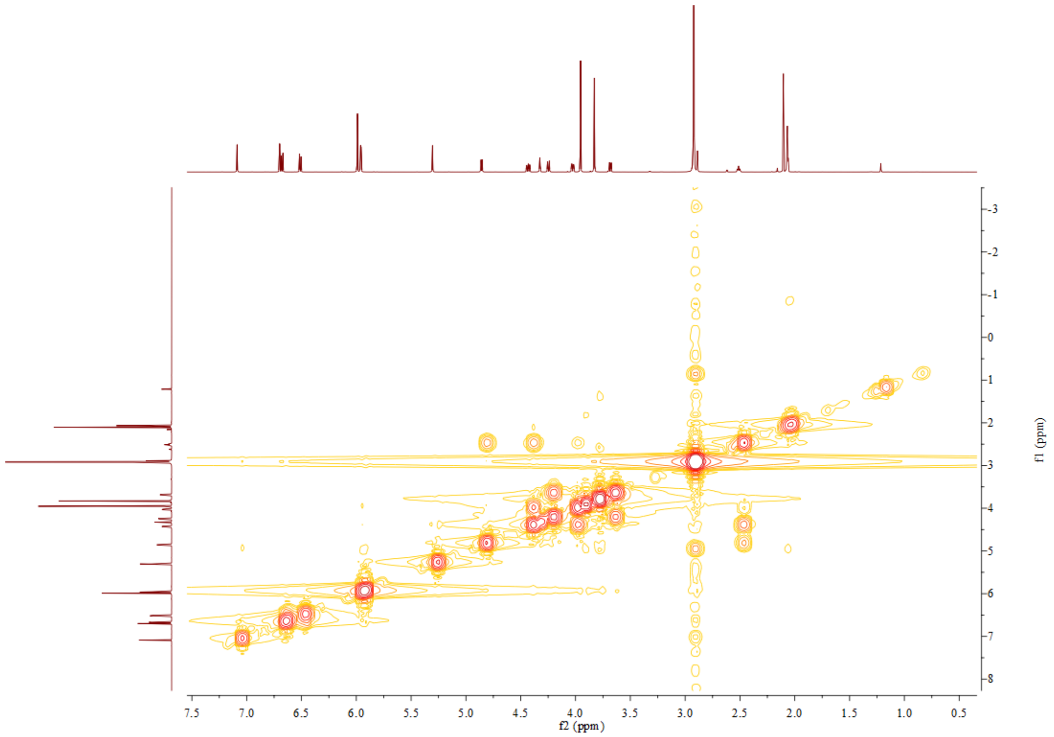

Figure S25. $^{1}H-^{1}H$ COSY spectrum of leptolignan C (**9**) in acetone-d_6.

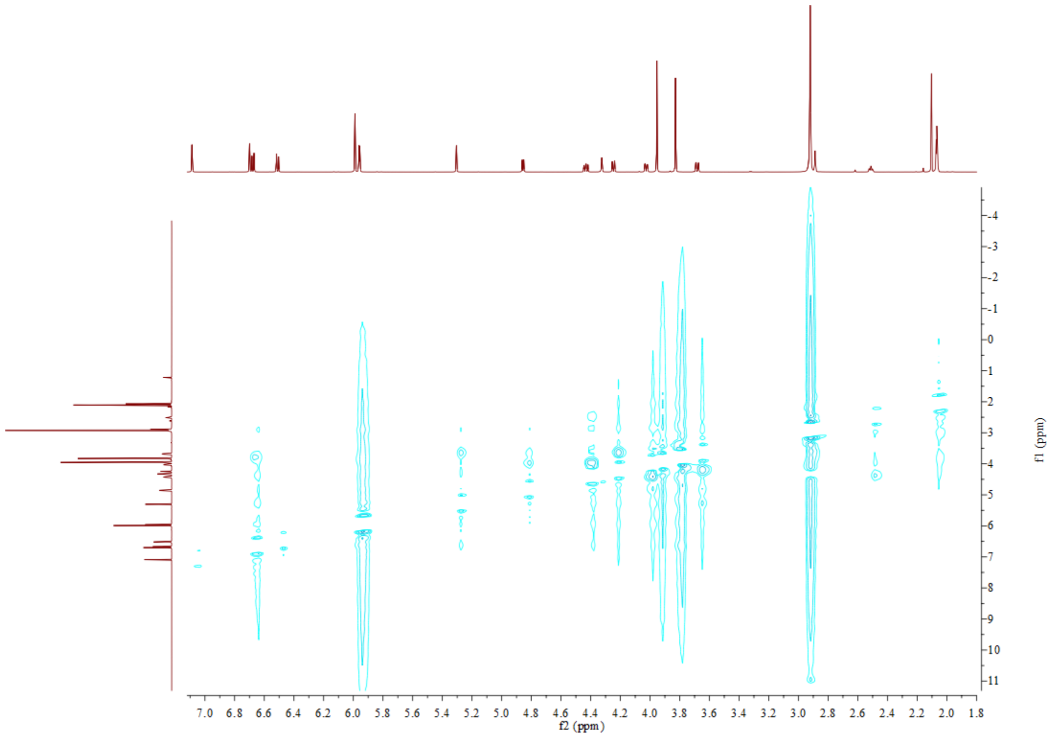

Figure S26. $^{1}H-^{1}H$ ROESY spectrum of leptolignan C (**9**) in acetone-d_6.

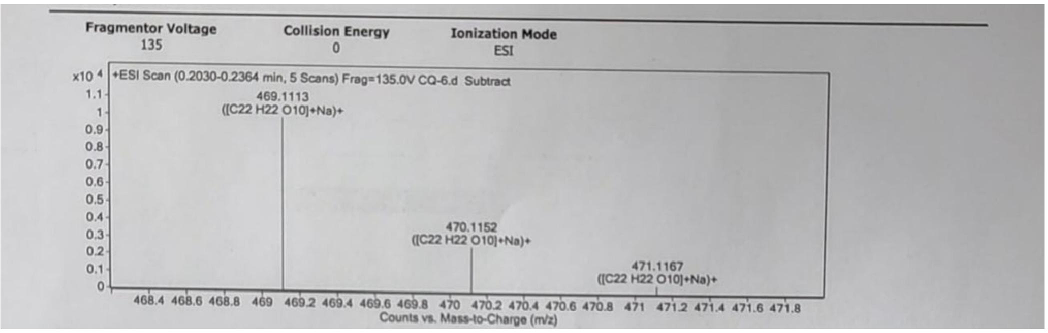

Figure S27. HR-ESI-MS spectrum of leptolignan C (**9**).

Formula	CalculatedMass	CalculatedMz	Mz	Diff. (mDa)	Diff. (ppm)	DBE
$C_{22}H_{22}O_{10}$	446.1213	469.1105	469.1113	-0.80	-1.71	12.0000

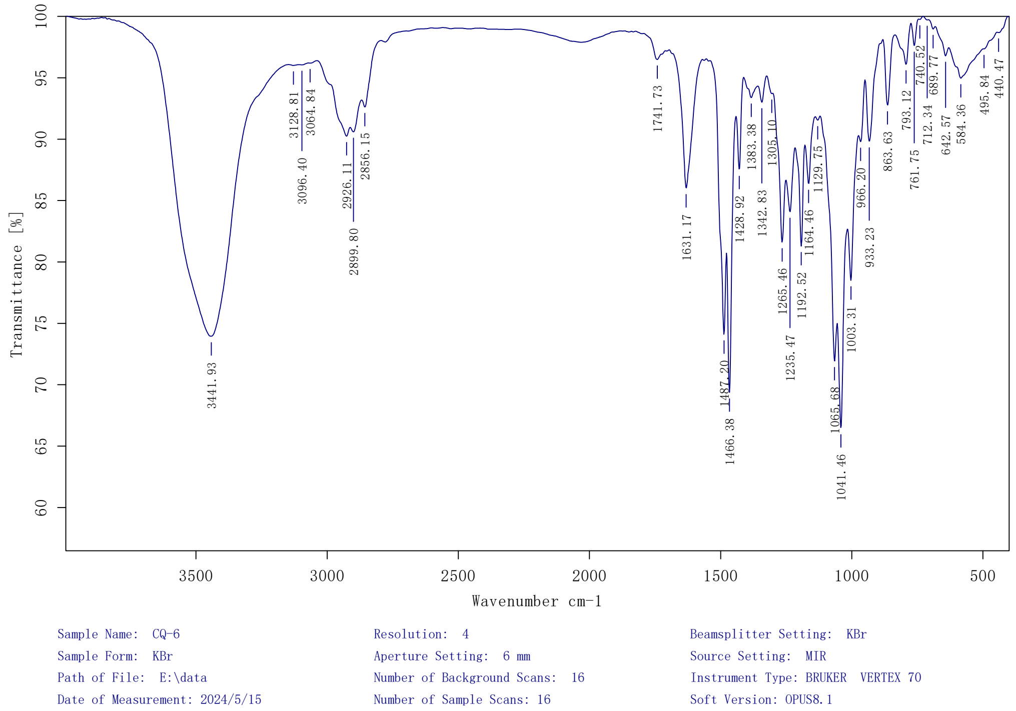

Figure S28. IR spectrum of leptolignan C (**9**).

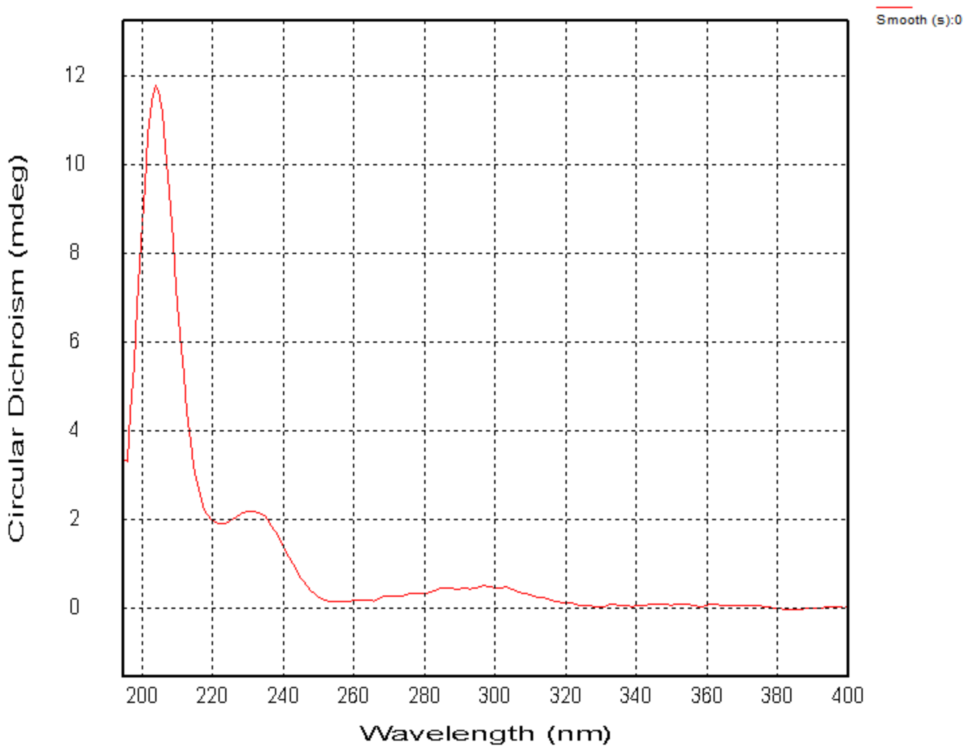

Figure S29. CD spectrum of leptolignan C (**9**).

n	Average	Std.Dev.	% RSD	Maximum	Minimum
5	167.20	0.45	0.26	168.00	167.00

S.No	Sample ID	Time	Result	Scale	OR °Arc	WLG.nm	Lg.mm	Conc.g/100ml	Temp.
1	CQ-6	11:01:03 AM	168.00	SR	0.168	589	100.00	0.100	25.0
2	CQ-6	11:01:09 AM	167.00	SR	0.167	589	100.00	0.100	25.0
3	CQ-6	11:01:16 AM	167.00	SR	0.167	589	100.00	0.100	25.0
4	CQ-6	11:01:22 AM	167.00	SR	0.167	589	100.00	0.100	25.0
5	CQ-6	11:01:28 AM	167.00	SR	0.167	589	100.00	0.100	25.0

Figure S30. Optical rotation data of leptolignan C (**9**).

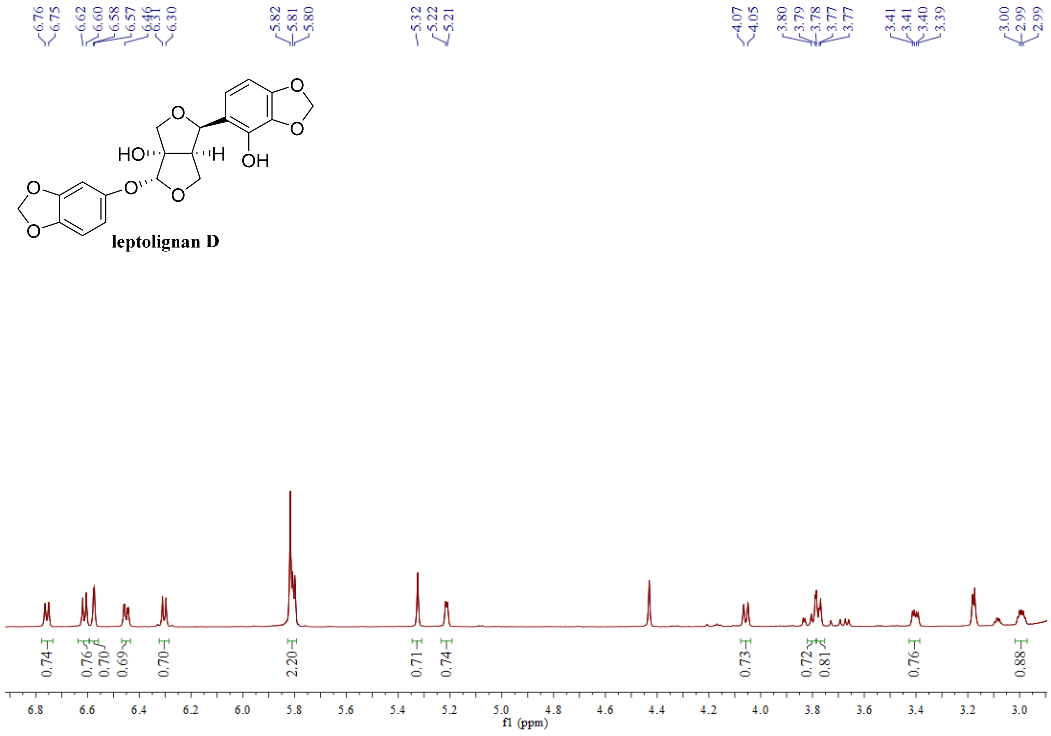

Figure S31. ^{1}H NMR spectrum of leptolignan D (**10**) recorded at 600 MHz in acetone-d_6.

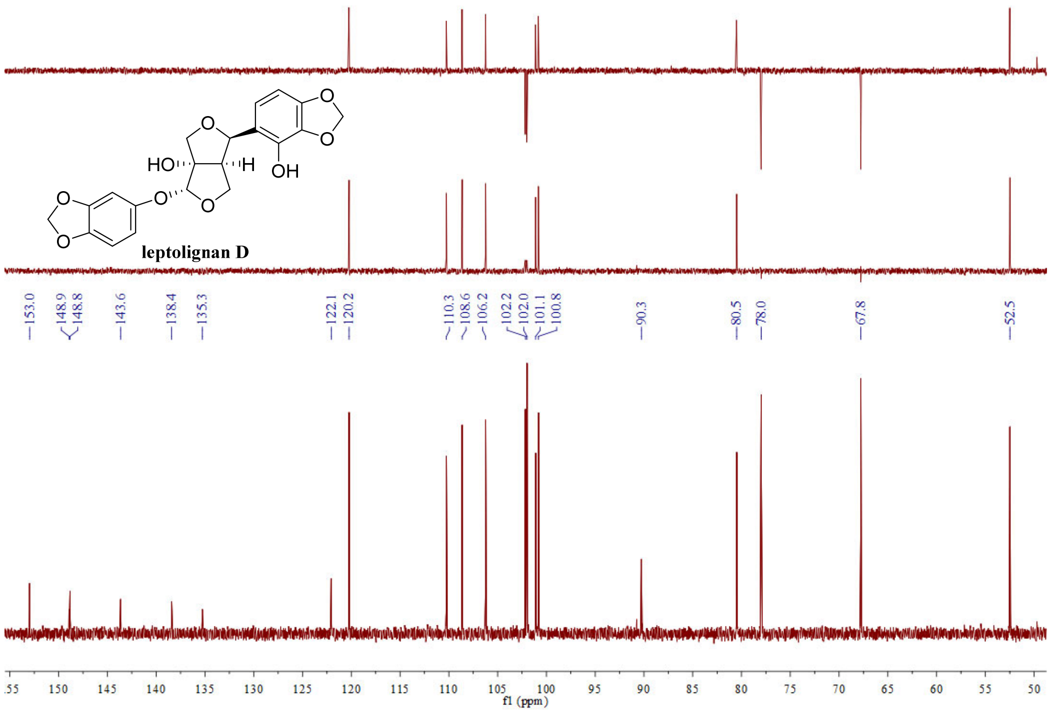

Figure S32. ^{13}C NMR and DEPT spectra of leptolignan D (**10**) recorded at 150 MHz in acetone-d_6.

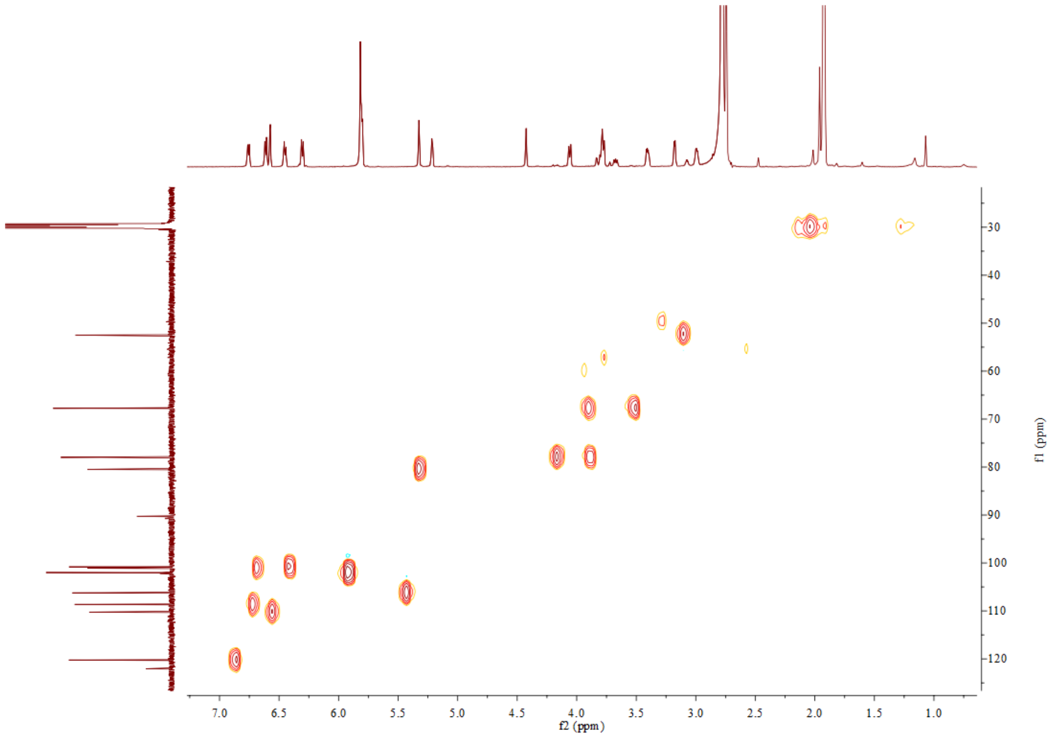

Figure S33. HSQC spectrum of leptolignan D (**10**) in acetone-d_6.

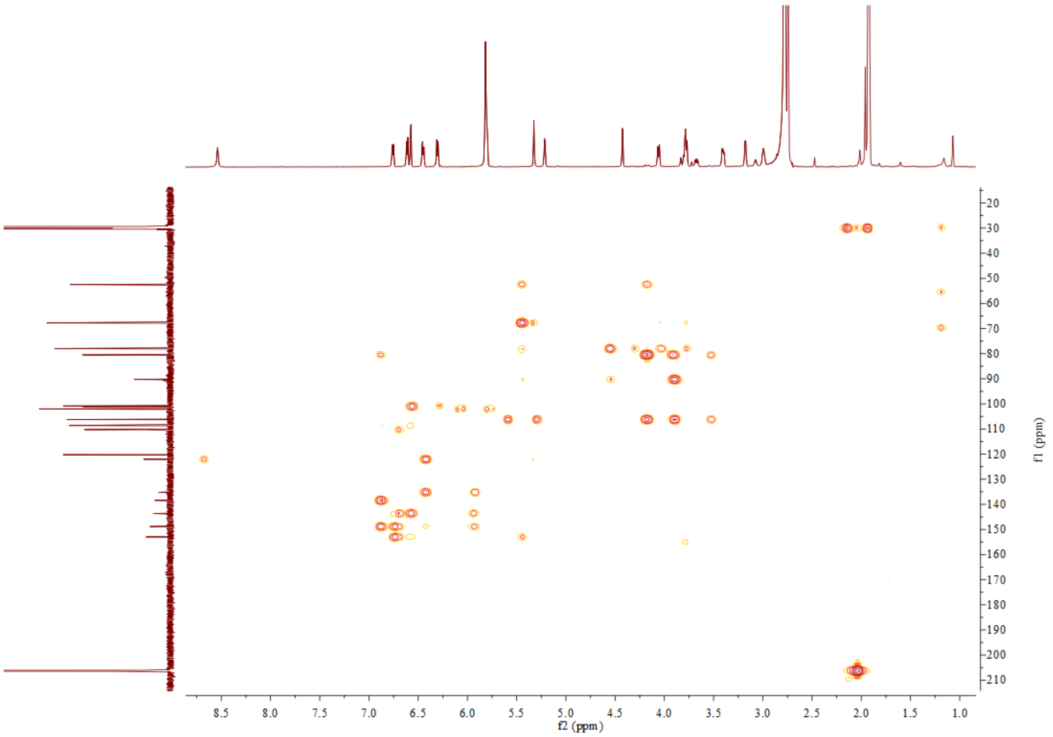

Figure S34. HMBC spectrum of leptolignan D (**10**) in acetone-d_6.

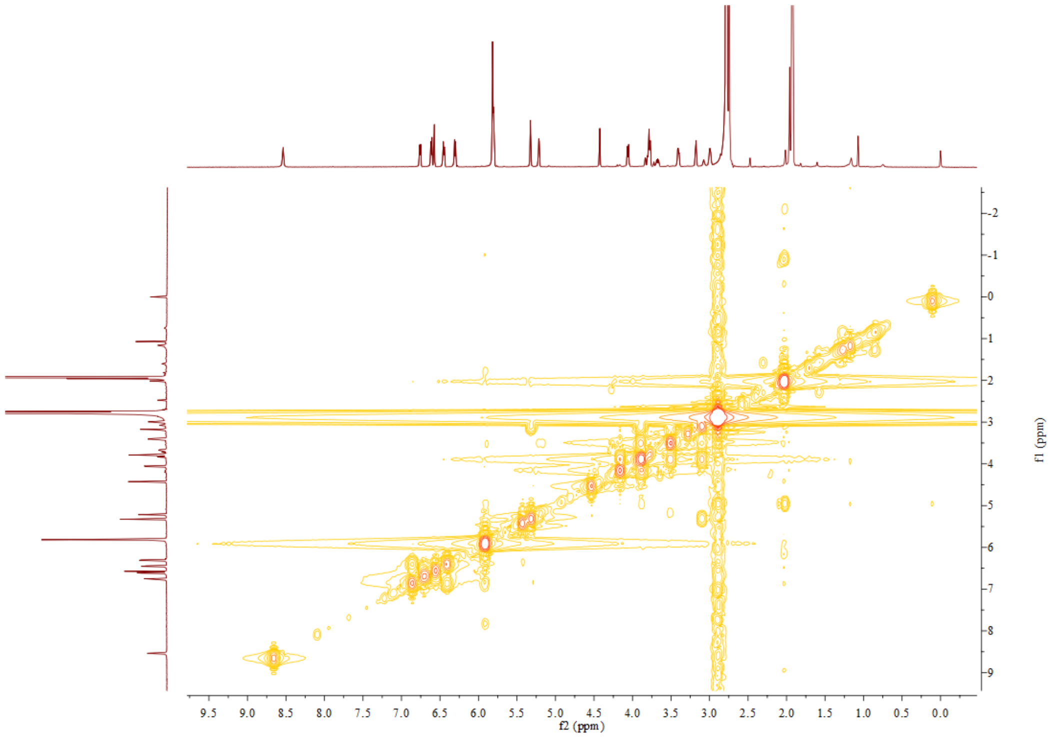

Figure S35. ^{1}H–^{1}H COSY spectrum of leptolignan D (**10**) in acetone-d_6.

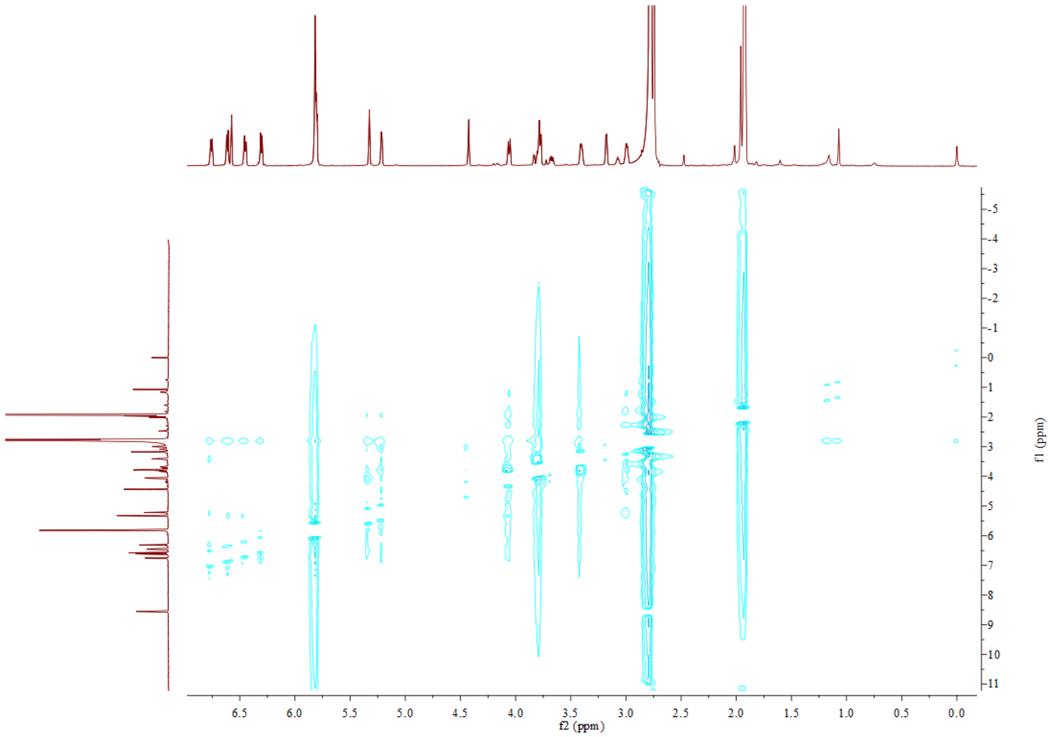

Figure S36. ^{1}H–^{1}H ROESY spectrum of leptolignan D (**10**) in acetone-d_6.

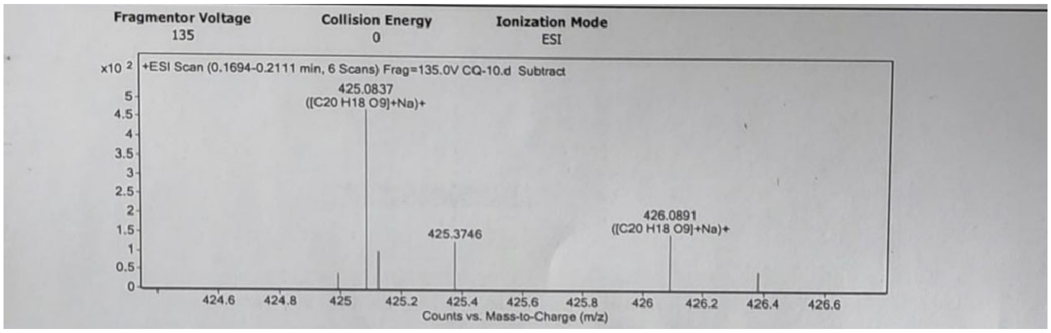

Figure S37. HR-ESI-MS spectrum of leptolignan D (**10**).

Formula	CalculatedMass	CalculatedMz	Mz	Diff. (mDa)	Diff. (ppm)	DBE
$C_{20}H_{18}O_9$	402.0951	425.0843	425.0837	0.60	1.41	12.0000

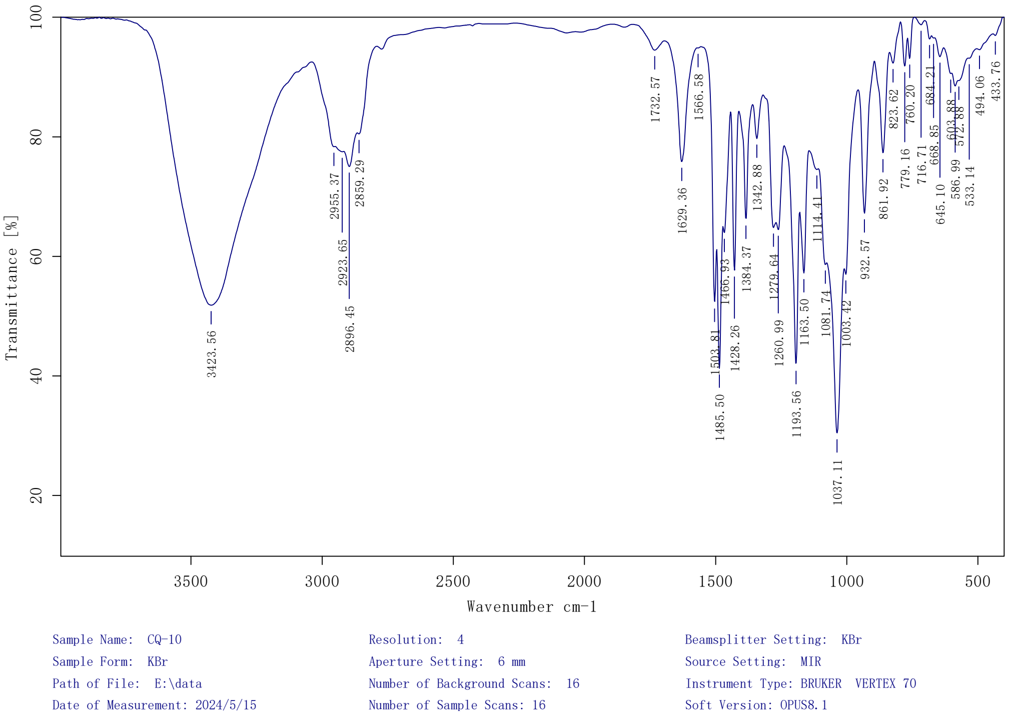

Figure S38. IR spectrum of leptolignan D (**10**).

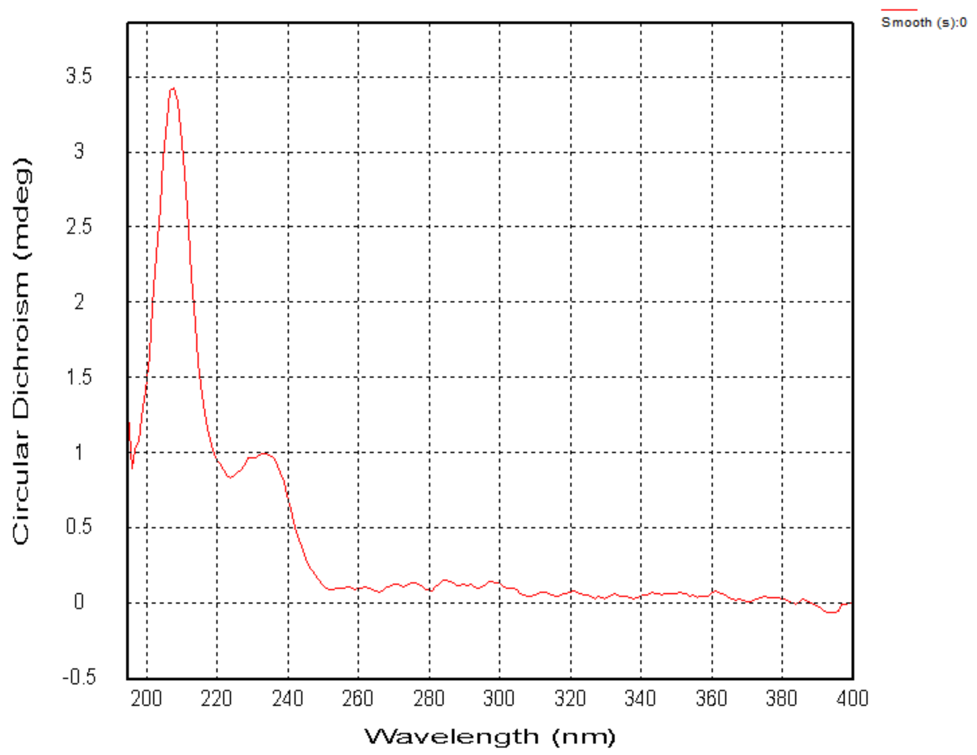

Figure S39. CD spectrum of leptolignan D (**10**).

n	Average	Std.Dev.	% RSD	Maximum	Minimum
5	3.00	0.00	0.00	3.00	3.00

S.No	Sample ID	Time	Result	Scale	OR °Arc	WLG.nm	Lg.mm	Conc.g/100ml	Temp.
1	CQ-10	01:42:46 PM	3.00	SR	0.003	589	100.00	0.100	25.0
2	CQ-10	01:42:52 PM	3.00	SR	0.003	589	100.00	0.100	25.0
3	CQ-10	01:42:59 PM	3.00	SR	0.003	589	100.00	0.100	25.0
4	CQ-10	01:43:05 PM	3.00	SR	0.003	589	100.00	0.100	25.0
5	CQ-10	01:43:11 PM	3.00	SR	0.003	589	100.00	0.100	25.0

Figure S40. Optical rotation data of leptolignan D (**10**).
